# Longitudinal trajectories of muscle impairments in growing boys with Duchenne muscular dystrophy

**DOI:** 10.1371/journal.pone.0307007

**Published:** 2025-03-18

**Authors:** Ines Vandekerckhove, Marleen Van den Hauwe, Tijl Dewit, Geert Molenberghs, Nathalie Goemans, Liesbeth De Waele, Anja Van Campenhout, Friedl De Groote, Kaat Desloovere

**Affiliations:** 1 Department of Rehabilitation Sciences, KU Leuven, Leuven, Belgium; 2 Department of Child Neurology, University Hospital Leuven, Leuven, Belgium; 3 Clinical Motion Analysis Laboratory, University Hospital Leuven, Pellenberg, Belgium; 4 Interuniversity Institute for Biostatistics and Statistical Bioinformatics (I-BioStat), KU Leuven, Leuven, Belgium; 5 Interuniversity Institute for Biostatistics and Statistical Bioinformatics (I-BioStat), Data Science Institute, Hasselt University, Hasselt, Belgium; 6 Department of Development and Regeneration, KU Leuven, Leuven, Belgium; 7 Department of Orthopedics, University Hospital Leuven, Leuven, Belgium; 8 Department of Movement Sciences, KU Leuven, Leuven, Belgium; University of Miami Miller School of Medicine: University of Miami School of Medicine, UNITED STATES OF AMERICA

## Abstract

**Background:**

Insights into the progression of muscle impairments in growing boys with Duchenne muscular dystrophy (DMD) remain incomplete due to the frequent oversight of normal maturation as a confounding factor, thereby restricting the delineation of sole pathological processes.

**Objective:**

To establish longitudinal trajectories for a comprehensive integrated set of muscle impairments, including muscle weakness, contractures and muscle size alterations, while correcting for normal maturation, in DMD.

**Methods:**

Thirty-three boys with DMD (aged 4.3–17 years) were included. Fixed dynamometry, goniometry, and 3D freehand ultrasound were used to repeatedly assess lower limb muscle strength, passive range of motion (ROM) and muscle size, resulting in 161, 178 and 64 assessments for the strength, ROM and ultrasound dataset, respectively. To account for natural strength development, ROM reduction, and muscle growth in growing children, muscle outcomes were converted to unit-less z-scores calculated in reference to typically developing (TD) peers. This allows the interpretation of the muscle outcomes as deficits or alterations with respect to TD. Mixed-effect models estimated the longitudinal change in muscle impairments.

**Results:**

At 4.3–4.9 years of age, all muscle strength outcomes and several ROMs (i.e., dorsiflexion, hamstrings, and hip extension) showed deficits relative to TD, while m. medial gastrocnemius size was increased. Most muscle outcomes remained stable or slightly improved until the ages of 6.6–9.4 years (except knee flexion strength). After this period, muscle strength (−0.27 to −0.45 z-score/year; p < 0.0044), dorsiflexion ROM (−0.23 to −0.33 z-score/year; p < 0.0007), m. medial gastrocnemius size (−0.56 z-score/year; p = 0.0022), and m. rectus femoris size (−0.36 z-score/year; p = 0.0054) declined.

**Conclusions:**

The current study established longitudinal trajectories of muscle impairments in boys with DMD. The results provided enriched history data and revealed promising outcome measures that could enhance the detection of the efficacy of novel therapeutic strategies. Future studies are necessary to validate these outcomes.

## Introduction

Duchenne muscular dystrophy (DMD) is an X-linked progressive muscular disorder, affecting two per 10,000 newborn boys [1]. Dystrophin deficiency leads to muscle cell membrane fragility and vulnerability to contraction-induced damage [2–4]. Progressive muscle degeneration then emerges with loss of contractile tissue and replacement by fat and fibrotic tissue [5,6]. Boys with DMD exhibit progressive muscle impairments, including muscle size alterations, weakness and contractures [5,7–9]. These muscle impairments contribute to alterations in posture and gait, reflected in a motor function decline with loss of ambulation between the ages of 7.1 and 18.6 years (mean age: 12.7 years) [5,8,10]. The state-of-the-art standard of care has altered the natural disease course and has increased life expectancy in DMD [5,11–14]. One of the treatment goals in DMD is prolonging ambulation [5,8]. Promising novel therapeutic strategies could potentially slow down disease progression and delay loss of ambulation. However, efforts to describe history data and develop outcome measures are urgently needed to overcome the difficulties in their clinical development [15–18]. To achieve this, establishing longitudinal trajectories of muscle impairments is essential, as it will enrich history data and could potentially reveal promising outcome measures.

Progressive muscle weakness is the primary clinical symptom in DMD. Significant weakness, evaluated through dynamometry, has been observed already in young boys with DMD and the strength deficit relative to typically developing (TD) children increased further with age [19–21]. This was primarily the result of the large age-related increase in muscle strength in TD, while in DMD absolute muscle strength remained relatively constant between the ages of 5 and 14 years [19,21]. After accounting for growth by normalizing muscle strength to body surface area or body mass, a significant reduction in strength with age was detected in boys with DMD [19,20]. Additionally, the use of more complex statistical models, such as non-linear piecewise models, showed that absolute muscle strength increased or remained stable until 8.5 years and then declined with age [22]. A previous longitudinal analysis even indicated that absolute knee extension/flexion strength increased until the age of 7.5 years, while after the age of 7.5 years strength declined, with even a more pronounced decline after 9 years of age [22]. Recently, Leon et al. [23] generated percentile curves of muscle strength for boys with DMD aged 4 to 18 years based on an extended longitudinal dataset consisting of 230 assessments in 73 boys. Knee extension strength exhibited the most pronounced decline, followed by hip flexion strength, and then knee flexion strength. A recent 8-year longitudinal study by Buckon et al. [24] delineated the progressive muscle weakness in boys with DMD (aged 4 to 15 years at baseline) and found that knee extension strength, followed by hip extension strength, declined the fastest.

Apart from progressive muscle weakness, other impairments also occur in boys with DMD. Due to fat and fibrotic tissue infiltration into the muscles, prolonged static positioning, and muscle weakness, boys with DMD experience progressive loss of passive range of motion (ROM), resulting in the development of contractures [7]. In general, lower limb contractures are strongly related to wheelchair reliance, with slow contracture evolution prior to loss of ambulation and rapid contracture evolution following loss of ambulation [25–27]. However, Choi et al. [27] found that 52.6% of ambulatory boys with DMD had ankle plantar flexion contractures and Willcocks et al. [25] showed that ROM in boys with DMD was significantly lower than in TD children, starting 5 years before loss of ambulation at the ankle, 2 years before at the knee, and 4 years before at the hip. Most studies have focused on the frequency and severity of contractures, while the progression rate of decreasing ROM has primarily been quantified for the ankle ROM, with limited investigation into the hip and knee ROMs. For example, Kiefer et al. [28] found that ankle dorsiflexion ROM decreased with 1.4° per year based on a longitudinal dataset of 1761 assessments in 322 boys with DMD. Furthermore, Leon et al. [23] generated percentile curves for ankle dorsiflexion ROM in boys with DMD, showing negative ROM values (deficit to reach neutral) from the age of 8 years with the knee extended and from the age of 9 years with the knee flexed, indicating pathological shortening of the ankle plantar flexors. However, previous studies did not account for the typical reduction in ROM observed in TD children aged 2 to 17 years [29–31].

The progressive loss of contractile tissue and proliferation of fat and fibrotic tissue result in varying muscle size alterations, such as profound atrophy in some muscles and (pseudo)hypertrophy in others [9]. (Pseudo)hypertrophy of the m. triceps surae is a well-known feature in DMD with reported similar contractile cross-sectional area (CSA) as in TD [9] but a total CSA up to 60% larger than in TD [19]. Yet, results on the change in m. triceps surae size with age in DMD are conflicting [9,19]. The m. quadriceps has been characterized by atrophy in DMD, with both smaller contractile and total CSA than in TD [9]. However, Mathur et al. [19] found hypertrophy of the m. quadriceps under the age of 10 years and atrophy from the age of 11 years. While the m. triceps surae and m. quadriceps represent two ends of a spectrum, the m. hamstrings and m. tibialis anterior presented with less distinctive muscle size alterations [9]. Magnetic resonance imaging (MRI) has been frequently used to quantify muscle size as well as composition and has repeatedly been suggested as a sensitive biomarker in DMD clinical trials [32–36]. Ultrasound has some advantages over MRI, since it is child-friendly, relatively cheap, fast, does not require sedation, and is clinically easily accessible because it can be collected using a portable device. Ultrasound studies showed that changes in muscle echo-intensity preceded motor regression and increased over time, highlighting the added value of ultrasound as a practical and child-friendly tool for estimating muscle composition and tracking disease progression longitudinally [37–39]. However, studies evaluating muscle size with ultrasound in DMD are scarce. Bulut et al. [40] reported increased muscle thickness of the m. vastus lateralis and m. medial gastrocnemius in children with DMD compared to TD children. However, Jansen et al. [38] and Pillen et al. [41] only found a reduced muscle thickness of the m. quadriceps at baseline without abnormalities in thickness of the other muscles. Morse et al. [42] even showed a decrease of 36% in the CSA of the m. medial gastrocnemius in adults with DMD compared to TD adults. Additionally, a longitudinal analysis indicated that lower limb muscle thickness normalized to body mass did not progress with age [38], while another study demonstrated larger absolute muscle thickness of the m. medial gastrocnemius in more severely affected boys than in less severely affected boys [40]. This highlights that the longitudinal evolution of muscle size alterations measured by ultrasound in growing boys with DMD is still unclear.

It can be concluded that the insights into the progression of muscle impairments in boys with DMD during growth remain incomplete. This inadequacy arises from the fact that not all lower limb muscle groups have undergone comprehensive assessment, and conflicting results among studies have been reported. Additionally, the confounding factor of normal maturation has frequently been overlooked, or various normalization techniques have been inconsistently applied. Therefore, it has been difficult to distinguish pathological changes from typical strength development, ROM reduction, and muscle growth observed in TD, hampering the interpretation of pure pathological processes during growth in DMD. We recently developed anthropometric-related TD percentile curves for muscle strength and size [43]. These curves enable us to convert individual muscle strength and size outcomes into unit-less z-scores, which indicate deficits relative to TD peers. Since typical strength development and normal muscle growth are accounted for in this way, the use of z-scores facilitates the interpretation of pathological alterations during growth. Similarly, the TD reference values of Mudge et al. [29] and Sankar et al. [30] for three age groups can be used to calculate unit-less z-scores for ROM, allowing to correct for the typical reduction in ROM. Therefore, the aim of this study was to establish longitudinal trajectories for a comprehensive integrated set of muscle impairments, including muscle weakness, contractures, and muscle size alterations, while correcting for normal maturation, in growing boys with DMD through a longitudinal follow-up design.

## Materials and methods

### Study design and participants

This longitudinal observational cohort study was designed with a protocol of repeated assessments with a variable number of assessments between participants and variable time intervals between assessments. In general, the time interval between repeated assessments was standardized at 6 months, except in cases of retrospective data, the impact of COVID-19, and for children whose condition was too mild to schedule a follow-up after 6 months.

Children with DMD were recruited via the multidisciplinary clinic of the Neuromuscular Reference Centre (NMRC) in the University Hospital Leuven campus Gasthuisberg between 22 June 2018 and 31 December 2022. Boys aged between 3 and 16 years at baseline, ambulatory for at least 100 meters (to enable the conduct of a 3D gait analysis as part of a larger project), and with a confirmed genetic diagnosis of DMD, were included. Cognitive and behavioral disorders preventing accurate measurements, a clinical picture of Becker muscular dystrophy, and history of muscle lengthening surgery were exclusion criteria. Use of corticosteroids and participation in clinical trials were permitted, as corticosteroid intake is part of the current state-of-the-art clinical care and a large portion of the children participated in clinical trials at the involved center. At the University Hospital Leuven, a proactive/preventive policy was implemented to address contractures. Hereto, nighttime ankle foot orthoses (AFOs) were initiated before any contractures occurred, often in conjunction with the start of corticosteroids. Occasionally, serial casting was also employed to manage early losses in ankle dorsiflexion ROM.

Retrospective data from the included children collected before 14 June 2018 (between 20 May 2015 and 14 June 2018) was accessed through the database of University Hospital Leuven between 22 June 2018 and 31 December 2022.

Thirty-three boys with DMD, aged between 4.3 and 17 years old, were repeatedly measured between 2015 and 2022 at multiple time points (1 to 11) with a variable time interval of 5-35 months, covering a follow-up period of 6 months to 6.6 years. In total, the collected dataset consisted of 181 observations (S1 Fig).

The local ethics committee (Ethical Committee UZ Leuven/KU Leuven; S61324) approved this study under the Declaration of Helsinki. All methods were performed in accordance with the relevant guidelines and regulations. The parents or caregivers of the participants signed a written informed consent and participants aged 12 years or older signed a written informed assent.

### Data collection and analysis

The same assessor performed all measurements on a specific patient, with the entire database being collected by two assessors: one who collected all the retrospective data and one who collected all the prospective data.

#### Anthropometry.

Body mass, height and lower limb segment lengths of the boys with DMD were measured at each follow-up session.

#### Muscle weakness.

Muscle strength was collected unilaterally to avoid fatigue and ensure participant cooperation. The weakest side, based on manual muscle testing, was selected as the assessed side to avoid mixing data from both the weakest and strongest sides across participants. We focused on the weakest side, as it may have a greater impact on overall functioning and is therefore more clinically relevant. If no weakest side could be identified, either due to similar outcomes or variability in manual muscle testing results between the two sides, preventing the designation of an overall weaker side, the assessed side was randomly chosen by flipping a coin, as this method is quick, easy, and free of bias.

A reliable and valid instrumented strength assessment was used to evaluate muscle weakness of the hip extensors, hip flexors, hip abductors, knee extensors, knee flexors, plantar flexors, and dorsiflexors [44,45]. The participants performed maximal voluntary isometric contractions (MVIC) using a fixed dynamometer (MicroFet, Hogan Health Industries, West Jordan, UT USA) in a standardized test position with 60° of hip flexion, 30° of knee flexion, and the ankle in the neutral position (A detailed description is provided in S1 Appendix) [44,45]. Custom-written Matlab (The Mathworks Inc., Natick, M.A., R2021b) scripts were used to extract the maximal force (in Newton) per MVIC and to calculate the mean maximal joint torque (in Newton meter) per muscle group by multiplying the mean maximal force over one to three representative MVIC trials with the lever arm, which was determined at 75% of the segment length (S1 Appendix). To account for typical strength development, anthropometric-related TD percentile curves for muscle strength (n = 153) were used to convert mean maximal joint torques into unit-less z-scores (S2 Fig) [43]. Hence, these z-scores reflect muscle strength deficits with respect to TD peers.

#### Contractures.

Contractures were assessed bilaterally, but only the values from the side selected for the strength assessment were included in further analyses to ensure consistency with the other measurements. During a standardized clinical examination, goniometry was used to measure the passive ROM of hip extension (modified Thomas test [29]), hip adduction (with an extended hip and knee on the assessed leg and 90° of hip and knee flexion on the contralateral leg [30]), knee extension [29], hamstrings (true popliteal angle [29]), and ankle dorsiflexion (with knee extended and knee flexed in 90° [29]) (A detailed description is provided in S2 Appendix). Previous studies reported acceptable intra-rater and inter-rater reliability of these measures [29]. Passive ROM was measured in degrees. Since TD percentile curves for ROM are currently lacking, the age-related normative reference values of Mudge et al. [29] were used to convert ROM measures into unit-less z-scores, accounting for the typical reduction in passive ROM during growth. Thereby, the normative mean of the corresponding age group (4–7 years, 8–11 years and 12–16 years) was subtracted from the individual ROM and then this difference was divided by the standard deviation of the total normative group. We decided to use the standard deviation of the total normative group, since the large difference in standard deviations between normative age groups would result in unrealistic changes in z-scores over time. A similar approach was used to calculate z-scores for hip adduction ROM, but based on the reference values for three age groups provided by Sankar et al. [30], since these were lacking in the study of Mudge et al. [29]. By calculating the z-scores based on data for three age groups, continuous changes with age were not fully controlled for, influencing the validity of z-scores. Therefore, both absolute ROM (in degrees) and unit-less ROM (in z-scores) were reported.

#### Muscle size alterations.

Muscle size was collected unilaterally on the selected side to reduce the overall duration of the measurement session. The reliable 3D freehand ultrasound (3DfUS) was used to assess muscle size alterations of the m. rectus femoris, m. medial gastrocnemius and m. tibialis anterior (A detailed description is provided in S3 Appendix) [46,47]. The orientation and position of 2D ultrasound images were quantified using a motion tracking system (Optitrack V120:Trio, NaturalPoint Inc., Corvallis, Oregon, USA) that tracked four reflective markers on the linear probe of a Telemed-Echoblaster B-mode ultrasound device (Telemed-Echoblaster 128 Ext-1Z, with a 5.9-cm 10-MHz linear US transducer, Telemed Ltd., Vilnius, Lithuania). To limit muscle deformation, a custom-made gel pad (i.e., the portico), was attached on the ultrasound probe [48]. Data were collected and processed using STRADWIN software (version 6.0; Mechanical Engineering, Cambridge University, Cambridge, UK). The Euclidean distances between relevant anatomical landmarks were calculated to estimate muscle lengths (in mm). Mid-belly anatomical CSA (in mm^2^) was defined by one manually drawn transverse plane segmentation alongside the inner muscle border at 50% of the muscle length. We applied the same ultrasound settings and parameter definitions as reported in previous studies [47,49]. In some children, high muscle degeneration strongly reduced the visibility of the muscle border. In those children, a single 2D image was collected at the mid-belly. Real-time feedback from the ultrasound images and palpation of the leg were used to identify landmarks. Subsequently, a tape measure was used to determine the midpoint between landmarks (i.e., mid-belly). To account for typical muscle growth, anthropometric-related TD percentile curves for muscle CSA (n = 143) were used to convert CSAs into unit-less z-scores (S3 Fig) [43]. Hence, these z-scores reflect muscle size alterations with respect to TD peers.

### Statistical analysis

To model the longitudinal change in muscle impairments, (non-)linear mixed-effect models were used.

Mixed models were fitted per outcome, i.e., strength of the seven muscle groups (expressed as z-scores), six passive ROMs (absolute (in degrees) as well as unit-less (in z-scores)) and three muscle CSAs (expressed in z-scores). The mixed models consisted of a mean structure (i.e., fixed effects) to delineate the average trajectory and a covariance structure, induced by random effects, to describe the inter-subject variability. Age (expressed in years) at each repeated assessment represented the time effect and was selected as the fixed effect. Random effects were included to model the variability in starting point (i.e., random intercept) and the variability in trajectory (i.e., random slope) among the subjects. We applied the previously documented workflow to construct the mixed-effect models [50–52]. Since the explorations suggested linear trends interrupted by breaking points, non-linear piecewise models were allowed. Thereby, estimated values from the explorations were used as starting values and the presence of multiple breakpoints was investigated. A breakpoint was defined if the F-test detected a significant change between adjacent slopes, whilst ensuring a minimum of 10 data points per regression line. Moreover, a breakpoint was not interpreted as a strict age where a change occurs, but rather as the approximate age around which a change in rate occurs. Non-nested linear and non-linear piecewise models were compared using the Akaike information criterion (AIC). Since, overall, the piecewise models demonstrated the smallest AIC, these models were selected. Yet, the difference with the linear models was small and we therefore documented the linear models in the supplementary materials (S4-S6 Figs and S1-S3 Tables). The piecewise model with observation *j* in subject *i* to estimate the outcome_ij_ was defined as follows:


$$Outcome_{ij}=\left(\alpha_{0}+a_{i0}\right)+\left(\beta_{1}+b_{i1}\right)*age_{ij}+\varepsilon_{ij}ifage_{ij}<\gamma_{1}$$

$$Outcome_{ij}=\left(\alpha_{0}+a_{i0}\right)+\gamma_{1}*\left(\left(\beta_{1}+b_{i1}\right)-\left(\beta_{2}+b_{i2}\right)\right)+\left(\beta_{2}+b_{i2}\right)*age_{ij}+\varepsilon_{ij}if\gamma_{1}\leq age_{ij}<\gamma_{2}$$

$$\begin{array}{l} Outcome_{ij}=\left(\alpha_{0}+a_{i0}\right)+\gamma_{1}*\left(\left(\beta_{1}+b_{i1}\right)-\left(\beta_{2}+b_{i2}\right)\right)+\gamma_{2}*\left(\left(\beta_{2}+b_{i2}\right)-\left(\beta_{3}+b_{i3}\right)\right)\\ +\left(\beta_{3}+b_{i3}\right)*age_{ij}+\varepsilon_{ij}if\gamma_{2}\leq age_{ij} \end{array}$$

with α_0_ =  intercept; a_i0_ =  random intercept; β_1_ =  regression slope if age_ij_ < γ_1_; b_i1_ =  random slope of β1; γ_1_ =  first breakpoint; β_2_ =  regression slope if γ_1_ ≤  age_ij_ < γ_2_; b_i2_ =  random slope of β_2_; γ_2_ =  second breakpoint; β_3_ =  regression slope if γ_2_ ≤  age_ij_; b_i3_ =  random slope of β_3_; ε_ij_ =  measurement error.

F-tests were performed to investigate if the intercepts, regression slopes, and breakpoints differed from zero. The significance threshold was set to α = 0.05. So-called empirical Bayes estimates were calculated after model formulation and used to detect outliers, i.e., subjects with an exceptional starting point and/or evolution with age. Data of outliers were checked. SAS® (version 9.4, Statistical Analysis Software, SAS Institute Inc., Cary, NC, USA) was used to conduct all analyses and visualizations.

## Results

After data collection and processing, quality check, and outliers removal, the strength dataset consisted of 161 observations in 31 boys, the ROM dataset of 178 observations in 32 boys, and the ultrasound dataset of 64 observations in 32 boys (S1 Fig). Group demographics are presented in Table 1, while the medical and clinical backgrounds for each participant are shown in S7 Fig and are detailed in S4 Table. The ultrasound dataset was smaller than the other datasets because of the following reasons. In the initial phase of the study (primarily involving retrospective data and the first measurements of the prospective data), ultrasound data was either not yet collected or the ultrasound technique was not fully refined. After this initial period, ultrasound data collection became systematic. However, due to the manual processing burden—particularly the challenges in identifying muscle borders in dystrophic muscles—the decision was made to process only a subset of the dataset. Instead of processing all available data, we selected ultrasound measurements at 1-year or 2-year intervals for processing, regardless of the ages of the participants or the stage of the disease progression.

**Table 1:** Group demographics.

Baseline characteristics	
Subjects (n)	33 in total database: 31 in strength dataset; 32 in ROM dataset and 32 in ultrasound dataset
Median age (Q1–Q3)	7.6 years (5.1–9.9)
Median body mass (Q1–Q3)	22.4 kg (18.6–32.5)
Median height (Q1–Q3)	1.16 m (1.1–1.27)
Median BMI (Q1–Q3)	17.1 kg/m^2^ (15.5–18.9)
Genotype	
Deletion	75.8%
Duplication	15.2%
Nonsense Point mutation	9.1%
Corticosteroids	
Daily Deflazacort (% subjects)	81.8%
Vamorolone (% subjects)	/
No steroids (% subjects)	18.2%
Participation in clinical trial with disease modifying medication (% subjects)	21.2%
Adherence nighttime ankle foot orthoses (% subjects)	84.9%
Serial casting (% subjects)	3.0%
**Characteristics over all observations**	
Subjects (n)	33
Observations (n)	181
Participants with certain number of assessments (n)	
1 assessment	1
2 assessments	5
3 assessments	3
4 assessments	6
5 assessments	4
6 assessments	5
7 assessments	0
8 assessments	3
9 assessments	1
10 assessments	1
11 assessments	4
Median age (Q1–Q3)	10.2 years (7.8–12.5)
Median body mass (Q1–Q3)	30.4 kg (22.5–40.3)
Median height (Q1–Q3)	1.23 m (1.14–1.31)
Median BMI (Q1–Q3)	18.9 kg/m^2^ (16.9–24.1)
Corticosteroids	
Daily Deflazacort (% observations)	94.5%
Vamorolone (% observations)	2.2%
No steroids (% observations)	3.3%
Participation in clinical trial with disease modifying medication (% observations)	37.6%
Adherence nighttime ankle foot orthoses (% observations)	79.6%
Serial casting (% observations)	6.6%
Loss of ambulation within 1 year after last assessment (% subjects)	30.3%

BMI, body mass index; kg, kilogram; m, meter; n, number; Q1, first quartile; Q3, third quartile; ROM, range of motion.

### Muscle weakness

**Fig 1:**
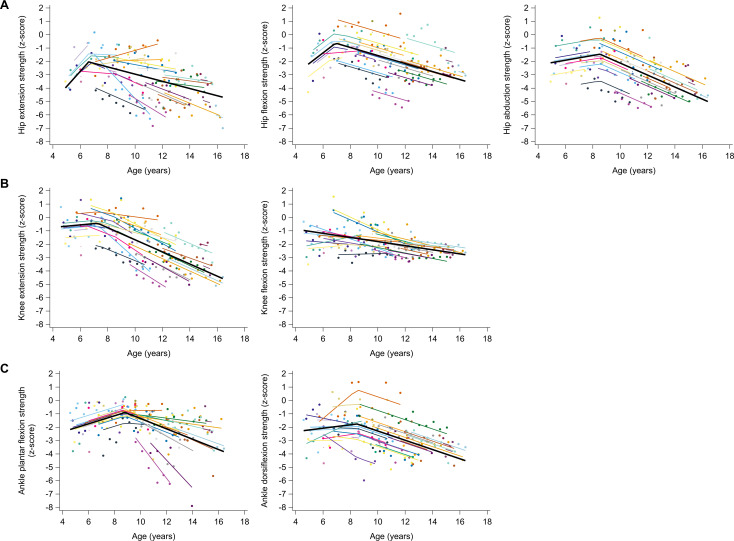
Predicted longitudinal trajectories for strength of hip muscles (A), knee muscles (B), and ankle muscles (C) with age in boys with DMD. The average predicted trajectory (thick black line), the individual predicted profiles (thinner colored lines), and the actual observed outcomes (colored dots) are displayed. Each color represents one patient with DMD. The estimates for the fixed effects are given in Table 2. DMD, Duchenne muscular dystrophy.

The longitudinal trajectories of muscle strength were characterized by piecewise trends, except for knee flexion strength (Fig 1 and Table 2). Hip extension strength increased with a rate of 1.14 z-score/year from a z-score of −4.0 at age 4.9 years to a z-score of −2.06 at age 6.6 years and then decreased with a rate of −0.27 z-score/year until a z-score of −4.71 at age 16.4 years. Hip flexion strength increased with 0.79 z-score/year from −2.20 z-score at age 4.9 years to −0.61 z-score at age 6.9 years and then decreased with −0.30 z-score/year until −3.46 z-score at age 16.4 years. Hip abduction strength was −2.10 z-score at age 4.9 years and remained constant until age 8.5 years. It then decreased with −0.45 z-score/year from −1.45 z-score at age 8.5 years to −4.99 z-score at age 16.4 years. Knee extension strength was −0.67 z-score at age 4.6 years and remained constant until age 7.3 years. It then decreased with −0.45 z-score/year from −0.40 z-score at age 7.3 years to −4.51 z-score at age 16.4 years. Knee flexion strength was −0.95 z-score at age 4.6 years and decreased with a rate of −0.15 z-score/year to −2.72 z-score at age 16.4 years. Ankle plantar flexion increased with a rate of 0.30 z-score/year from −2.18 z-score at age 4.6 years to −0.90 z-score at age 8.8 years and then decreased with −0.39 z-score/year until −3.86 z-score at age 16.4 years. Ankle dorsiflexion strength was −2.24 z-score at age 4.6 years and remained constant until age 8.5 years. It then decreased with −0.35 z-score/year from −1.74 z-score at age 8.5 years to −4.51 z-score at age 16.4 years. The differences in individual predicted trajectories for muscle strength between boys who participated in clinical trials and those who did not, as well as between boys who lost ambulation within one year after the last assessment and those who did not, are explored in S8 and S9 Figs, respectively.

**Table 2:** Fixed effects of piecewise models for the longitudinal trajectories of the muscle strength outcomes with age in boys with DMD.

Outcomes	n subj	n obs	Intercept	Regression coefficients (β) and breakpoints (γ)		
			α_0_ (CI)	β_1_ (CI)	γ_1_ (CI)	β_2_ (CI)
			*p-value*	*p-value*	*p-value*	*p-value*
Hip extension strength (z-score)	31	141	−9.60 (−13.84 −5.36)	1.14 (0.47 1.82)	6.6 (6.6 6.6)	−0.27 (−0.45 −0.09)
			***<0.0001***	***0.0017***	***<0.0001***	***0.0044***
Hip flexion strength (z-score)	31	141	−6.08 (−10.24 −1.92)	0.79 (0.11 1.46)	6.9 (6.1 7.7)	−0.30 (−0.42 −0.18)
			***0.0056***	***0.0242***	***<0.0001***	***<0.0001***
Hip abduction strength (z-score)	31	140	−2.98 (−5.02 −0.94)	0.18 (−0.11 0.47)	8.5 (7.2 9.8)	−0.45 (−0.58 −0.32)
			***0.0056***	*0.2228*	***<0.0001***	***<0.0001***
Knee extension strength (z-score)	31	161	−1.13 (−2.23 −0.03)	0.10 (−0.07 0.26)	7.3 (6.3 8.2)	−0.45 (−0.56 −0.34)
			***0.0453***	*0.2417*	***<0.0001***	***<0.0001***
Knee flexion strength (z-score)	31	161	−0.26 (−1.20 0.67)	−0.15 (−0.24 −0.07)		
			*0.5704*	***0.0011***		
Plantar flexion strength (z-score)	31	161	−3.55 (−5.46 −1.64)	0.30 (0.05 0.55)	8.8 (8.1 9.6)	−0.39 (−0.57 −0.20)
			***0.0007***	***0.0191***	***<0.0001***	***0.0002***
Dorsiflexion strength (z-score)	31	161	−2.84 (−4.88 −0.81)	0.13 (−0.14 0.39)	8.5 (8.5 8.5)	−0.35 (−0.45 −0.24)
			***0.0078***	*0.3339*	***<0.0001***	***<0.0001***

p-values in bold indicate significance level at p <  0.05.

The following symbols represent: α_0_ =  intercept; β_1_ =  regression slope if age < γ_1_; γ_1_ =  first breakpoint; β_2_ =  regression slope if γ_1_ ≤  age.

CI, 95% confidence interval; DMD, Duchenne muscular dystrophy; n, number; obs, observations; subj, subjects.

### Contractures

**Fig 2:**
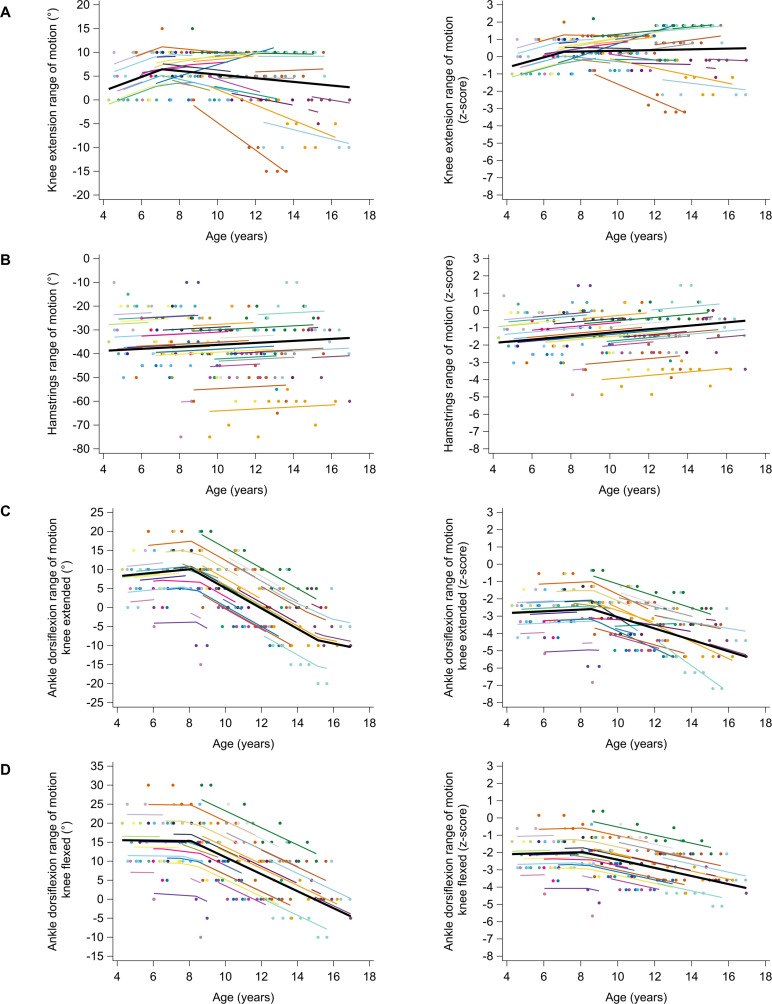
Predicted longitudinal trajectories for absolute (left column) and unit-less (right column) knee extension ROM (A), hamstrings ROM (B), ankle dorsiflexion with knee extended (C), and ankle dorsiflexion with knee flexed (D) with age in boys with DMD. The average predicted trajectory (thick black line), the individual predicted profiles (thinner colored lines), and the actual observed outcomes (colored dots) are displayed. Each color represents one patient with DMD. The estimates for the fixed effects are given in Table 3. DMD, Duchenne muscular dystrophy.

The longitudinal trajectories of the ROMs revealed a clear degeneration solely for ankle dorsiflexion (Fig 2 and Table 3). The absolute ankle dorsiflexion ROM with knee extended was 8.3° at age 4.3 years and remained constant until age 8.1 years. It then decreased with −2.7°/year from 10.1° at age 8.1 years to −8.6° at age 15.1 years, and lastly decreased with −1°/year until −10.4° at age 17 years. The unit-less ankle dorsiflexion ROM with knee extended was −2.82 z-score at age 4.3 years and remained constant until age 8.6 years. It then decreased with −0.33 z-score/year from −2.60 z-score at age 8.6 years to −5.36 z-score at age 17 years. The absolute ankle dorsiflexion ROM with knee flexed was 15.5° at age 4.3 years and remained constant until age 8.1 years. It then decreased with −2.2°/year from 15.3° at age 8.1 years to −4.5° at age 17 years. The unit-less ankle dorsiflexion ROM with knee flexed was −2.08 z-score at age 4.3 years and remained constant until age 8.1 years. It then decreased with −0.23 z-score/year from −1.97 z-score at age 8.1 years to −4.0 z-score at age 17 years. For hip extension and adduction ROMs, no reliable and valid mixed models could be fitted. The data exploration (S10 Fig) suggested, however, a subtle downward trend for the absolute hip extension and adduction ROMs. Absolute knee extension ROM increased with a rate of 1.5°/year from 2.3° at age 4.3 years to 6.4° at age 7.1 years and then remained constant until 2.7° at age 17 years. Unit-less knee extension ROMs increased with a rate of 0.29 z-score/year from −0.56 z-score at age 4.3 years to 0.26 z-score at age 7.1 years and then remained constant until 0.45 z-score at age 17 years. Absolute hamstrings ROM was −38.7° at age 4.3 years, but did not progress with age. Unit-less hamstrings ROM increased with 0.10 z-score/year from −1.85 z-score at age 4.3 years to −0.59 z-score at age 17 years. The differences in individual predicted trajectories for absolute and unit-less ROMs between boys who participated in clinical trials and those who did not, as well as between boys who lost ambulation within one year after the last assessment and those who did not, are explored in S11 and S12 Figs, respectively.

**Table 3:** Fixed effects of piecewise models for the longitudinal trajectories of the absolute and unit-less ROMs with age in boys with DMD.

Outcomes	n subj	n obs	Intercept	Regression coefficients (β) and breakpoints (γ)				
			α_0_ (CI)	β_1_ (CI)	γ_1_ (CI)	β_2_ (CI)	γ_2_ (CI)	β_3 _(CI)
			*p-value*	*p-value*	*p-value*	*p-value*	*p-value*	*p-value*
Knee extension ROM (°)	32	176	−4.02 (−9.16 1.12)	1.47 (0.71 2.24)	7.1 (7.1 7.1)	−0.38 (−0.89 0.12)		
			*0.1208*	***0.0004***	***<0.0001***	*0.1338*		
Knee extension ROM (z-score)	32	176	−1.80 (−2.84 −0.76)	0.29 (0.14 0.45)	7.1 (7.1 7.1)	0.02 (−0.08 0.12)		
			***0.0013***	***0.0006***	***<0.0001***	*0.6783*		
Hamstrings ROM (°)	32	176	−40.47 (−50.12 −30.83)	0.42 (−0.42 1.26)				
			***<0.0001***	*0.3263*				
Hamstrings ROM (z-score)	32	176	−2.28 (−3.21 −1.35)	0.10 (0.01 0.19)				
			***<0.0001***	***0.0239***				
Dorsiflexion ROM knee extended (°)	32	178	6.35 (−1.96 14.66)	0.46 (−0.76 1.68)	8.1 (6.9 9.3)	−2.66 (−3.42 −1.91)	15.1 (13.5 16.8)	−0.98 (−1.95−0.02)
			*0.1294*	*0.4482*	***<0.0001***	***<0.0001***	***<0.0001***	***0.0466***
Dorsiflexion ROM knee extended (z-score)	32	178	−3.03 (−4.44 −1.63)	0.05 (−0.14 0.24)	8.6 (8.6 8.6)	−0.33 (−0.51 −0.15)		
			***0.0001***	*0.5815*	***<0.0001***	***0.0007***		
Dorsiflexion ROM knee flexed (°)	32	178	15.77 (6.33 25.22)	−0.06 (−1.31 1.19)	8.1 (8.1 8.1)	−2.24 (−2.80 −1.67)		
			***0.0018***	*0.9221*	***<0.0001***	***<0.0001***		
Dorsiflexion ROM knee flexed (z-score)	32	178	−2.21 (−3.80 −0.63)	0.03 (−0.19 0.25)	8.1 (6.0 10.2)	−0.23 (−0.33 −0.14)		
			***0.0078***	*0.7914*	***<0.0001***	***<0.0001***		

p-values in bold indicate significance level at p <  0.05.

The following symbols represent: α_0_ =  intercept; β_1_ =  regression slope if age < γ_1_; γ_1_ =  first breakpoint; β_2_ =  regression slope if γ_1_ ≤  age < γ_2_; γ_2_ =  second breakpoint; β_3_ =  regression slope if γ_2_ ≤  age.

CI, 95% confidence interval; DMD, Duchenne muscular dystrophy; n, number; obs, observations; ROM, range of motion; subj, subjects.

### Muscle size alterations

**Fig 3:**
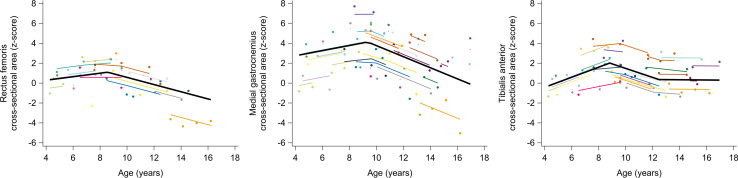
Predicted longitudinal trajectories for muscle size alterations with age in boys with DMD. The average predicted trajectory (thick black line), the individual predicted profiles (thinner colored lines), and the actual observed outcomes (colored dots) are displayed. Each color represents one patient with DMD. The estimates for the fixed effects are given in Table 4. DMD, Duchenne muscular dystrophy.

The longitudinal trajectories of muscle size alterations were characterized by piecewise trends (Fig 3 and Table 4). The m. rectus femoris CSA was 0.35 z-score at age 4.3 years and remained constant until age 8.6 years. It then decreased with −0.36 z-score/year from 1.12 z-score at age 8.6 years to −1.63 z-score at age 16.2 years. The m. medialis gastrocnemius CSA was 2.83 z-score at age 4.3 years and remained constant until age 9.4 years. It then decreased with −0.56 z-score/year from 4.15 z-score at age 9.4 years to −0.11 z-score at age 17 years. The m. tibialis anterior CSA increased with 0.51 z-score/year from −0.26 z-score at age 4.3 years to 2.06 z-score at age 8.8 years, then decreased with −0.45 z-score/year until 0.42 z-score at age 12.5 years, and lastly remained relatively constant until 0.37 z-score at age 17 years. The differences in individual predicted trajectories for muscle size alterations between boys who participated in clinical trials and those who did not, as well as between boys who lost ambulation within one year after the last assessment and those who did not, are explored in S13 and S14 Figs, respectively.

**Table 4:** Fixed effects of piecewise models for the longitudinal trajectories of the muscle size alterations with age in boys with DMD.

Outcomes	n subj	n obs	Intercept	Regression coefficients (β) and breakpoints (γ)				
			α_0_ (CI)	β_1_ (CI)	γ_1_ (CI)	β_2_ (CI)	γ_2_ (CI)	β_3 _(CI)
			*p-value*	*p-value*	*p-value*	*p-value*	*p-value*	*p-value*
Rectus femoris CSA (z-score)	23	44	−0.42 (−2.25 1.42)	0.18 (−0.09 0.44)	8.6 (6.9 10.2)	−0.36 (−0.60 −0.12)		
			*0.6426*	*0.1780*	***<0.0001***	***0.0054***		
Medial gastrocnemius CSA (z-score)	32	64	1.72 (−2.67 6.10)	0.26 (−0.31 0.82)	9.4 (7.7 11.0)	−0.56 (−0.90 −0.22)		
			*0.4307*	*0.3588*	***<0.0001***	***0.0022***		
Tibialis anterior CSA (z-score)	28	62	−2.44 (−4.77 −0.11)	0.51 (0.21 0.80)	8.8 (8.8 8.8)	−0.45 (−0.77 −0.13)	12.5 (10.8 14.2)	−0.01 (−0.26 0.24)
			***0.0408***	***0.0015***	***<0.0001***	***0.0075***	***<0.0001***	*0.9187*

p-values in bold indicate significance level at p <  0.05.

The following symbols represent: α_0_ =  intercept; β_1_ =  regression slope if age < γ_1_; γ_1_ =  first breakpoint; β_2_ =  regression slope if γ_1_ ≤  age < γ_2_; γ_2_ =  second breakpoint; β_3_ =  regression slope if γ_2_ ≤  age.

CI, 95% confidence interval; CSA, cross-sectional area; DMD, Duchenne muscular dystrophy; n, number; obs, observations; subj, subjects.

## Discussion

This prospective longitudinal study established comprehensive trajectories of a combined set of muscle impairments, consisting of muscle weakness, contractures, and muscle size alterations, in the same cohort of growing boys with DMD. We adjusted for typical strength development, ROM reduction and muscle growth observed in TD by calculating unit-less z-scores in reference to TD peers. The use of these unit-less z-scores allows for the comparison of pathological involvement between muscle groups and different muscle outcomes.

Muscle weakness in growing boys with DMD follows a non-linear longitudinal trajectory (except for knee flexion), illustrating that its progression is more complex than a continuous increase. Initially, muscle strength improved (for hip extension, hip flexion and plantar flexion) or remained stable (for hip abduction, knee extension and dorsiflexion) and then declined after 6.6–8.8 years, except for knee flexion. Lerario et al. also described muscle strength at the knee as piecewise trends with initial improvements before 7.5 years of age and declines after. The initial improvement or stable period is consistent with the commonly held concept of the “honeymoon” period in DMD [53,54]. However, our results indicated that boys with DMD are already weaker than TD children at young ages, which aligns with previous literature [19–21]. Among muscle groups, the largest initial deficit was observed in hip extension strength (+/− −4 z-score), followed by hip flexion, hip abduction and ankle muscle strength (+/− −2 z-score), while the smallest initial deficit was noted in knee muscle strength (+/− −1 z-score). This only partially conforms with the known pattern of proximal to distal muscle weakness in DMD, since, unexpectedly, the ankle muscles were initially weaker than the knee muscles. This discrepancy could be attributed to differences in standardized test positions between the current measurement protocol and those reported in previous literature. However, as TD children performed the MVIC under identical conditions, this alone does not fully account for the observed strength deficits. An additional explanation is that children with DMD may not yet be capable of executing the MVIC correctly around the ankle at a young age. This is further supported by the initial improvement in PF strength and the high variability in DF strength at young ages. These early deficits may reflect a maturation effect rather than true muscle weakness. This could account for the larger deficits observed around the ankle compared to the knee in children with DMD. After the initial improvement or stable period, the strength progression rate changed to a decline at a certain age (i.e., breakpoint). Among muscle groups, this age did increase according to the proximal to distal pattern of muscle weakness (with exception of hip abduction and knee flexion). Indeed, hip extension and flexion strength began to decline at an earlier age than knee extension strength, which was followed by ankle dorsiflexion and plantar flexion strength. Yet, the order of decline rates from steepest to least steep among muscle groups did not align with this pattern. Specifically, knee extension and hip abduction strength showed the steepest declines, followed by plantar flexion, dorsiflexion, hip flexion, hip extension, and knee flexion strength. The variations in decline rate among muscle groups can be partially attributed to the timing of the breakpoint and the existing initial weakness. Previous literature reported a similar sequence of decline rate among muscle groups [23,24]. However, the longitudinal trajectories are not entirely equivalent, but due to methodological discrepancies among the studies, comparing the results proves challenging. Our results showed a flattening of the deterioration for hip extension, hip flexion, hip abduction and knee extension strength through the linear models (S4 Fig and S1 Table). Such a ceiling effect has also been reported by previous literature, as muscles can only degenerate until a certain point [26,38].

The trajectories of the ROMs revealed a clear decline solely for ankle dorsiflexion. In DMD, ankle plantar flexion contractures are the first contractures to develop, and, hence, occur more frequently and severely than hip and knee contractures in the ambulatory period [26,27]. Our results indicated already important plantar flexion contractures at young ages, which further progressively increased after the age of 8 years. Larger deficits were observed for dorsiflexion ROM with knee extended than with knee flexed, suggesting that the m. gastrocnemius is more affected than the m. soleus in the boys with DMD. Previous literature found that the absolute dorsiflexion ROM declined linearly from early ages onwards [23,28], which is in contrast with the non-linear piecewise trajectories in the current results. Although the observed decline rates in absolute dorsiflexion ROM were steeper than the previously reported longitudinal results of Kiefer et al. [28], the boys with DMD in the current study lost the ability to achieve neutral ankle angles at approximately 12 years of age with knee extended and at approximately 14 years of age with knee flexed, which was at an older age than reported by Leon et al. [23]. The proactive/preventive policy of addressing contractures with the early use of nighttime AFOs and occasional serial casting may have played an important role in retaining dorsiflexion ROM during the initial stable phase and in delaying the inability to achieve neutral ankle angles. In contrast to the distal plantar flexion contractures, proximal lower extremity contractures develop slowly in the ambulatory period but tend to progress rapidly after transitioning to the wheelchair [7,26,27]. The data exploration of absolute hip extension ROM and, absolute and unit-less hip adduction ROMs suggested a subtle downward trend (S10 Fig). Hip extension ROM already showed deficits in young children, indicating early hip flexion contractures, and continued to decline at a rate similar to that observed in TD. Hip adduction ROM declined more rapidly than in TD children, although it did not exhibit large deficits initially, suggesting that hip abduction contractures are absent early on but may develop after loss of ambulation. Although knee extension ROM did not decline with age, and no average deficit was found with respect to TD, there was significant inter-subject variability. Notably, three patients presented with evident knee flexion contractures. Interestingly, these patients lost ambulation within one year after the last assessment (S12 Fig). Deficits in hamstrings ROM were observed in young children, reflecting hamstrings contractures early on. However, the ROM tended to slightly catch up to that of TD children.

The trajectories of the muscle size alterations were muscle-specific, which aligns with previous literature [9]. Our results indicated that the m. rectus femoris CSA decreased after the age of 8.6 years, leading to atrophy at older ages. This partially aligns with the cross-sectional findings of Mathur et al. [19], who observed hypertrophy of the m. quadriceps under the age of 10 years and atrophy from the age of 11 years. In contrast, our results indicated obvious hypertrophy in the m. medial gastrocnemius, however with a decrease from 9.4 years of age. (Pseudo)hypertrophy is a well-known symptom of DMD and has been repeatedly reported by previous studies [9,19,40]. To the best of our knowledge, Morse et al. [42] is the only study that reported atrophy of m. medial gastrocnemius, although this was observed in adults with DMD. Hence, our observed trajectory of the m. medial gastrocnemius CSA, i.e., decreasing hypertrophy, may result in atrophy at adult ages. Although the m. rectus femoris and m. medial gastrocnemius CSAs displayed similar longitudinal trajectories, both muscles were characterized by different pathological processes (i.e., m. rectus femoris atrophy vs m. medial gastrocnemius hypertrophy), a finding also reported by the cross-sectional study of Wokke et al. [9]. In line with the observations of Wokke et al. [9], the trajectory of m. tibialis anterior CSA appeared less clearly defined with a high inter-subject variability.

This study provided novel insights by combining a set of different muscle outcomes from multiple muscle groups as well as by expressing them as deficits or alterations in reference to TD. Overall, the pathological trajectories of most muscle outcomes with age followed a similar non-linear, piecewise pattern, characterized by an initial phase of improvement or stability lasting until 6.6-9.4 years, and a subsequent decline after these ages. This indicates that despite the progressive nature of DMD, the deterioration of muscle outcomes is more complex than a steady decline. There is a period in which there is no deterioration (i.e., honeymoon). Both the start of corticosteroids and the preventive use of nighttime AFOs may potentially play a significant role in this period of stability. However, all muscle strength outcomes and several ROMs, i.e., hip extension, hamstrings and ankle dorsiflexion ROMs, showed initial deficits in reference to TD at young ages, while m. medial gastrocnemius size was increased. These proximal contractures were already present in young boys with DMD, but did not further increase with age during the ambulatory period. Conversely, general muscle weakness and plantar flexion contractures were the most important muscle impairments that exhibited the steepest increases after the initial period of stability, resulting in large deficits at older ages. Moreover, the longitudinal trajectories of deficits in muscle outcomes appeared to be coupled. As such, it is interesting to note the decline in muscle strength, ROM and CSA around the ankle, all of which began around 8–9 years of age.

Although this study improved the insights into the longitudinal trajectories of muscle impairments in DMD, there were some limitations. The children with DMD enrolled in the study at different ages and the baseline age range was wide. Additionally, the boys with DMD were repeatedly measured at different time points with variable time interval and variable number of repeated assessments. A limited number of repeated assessments was collected in some of the children with DMD. However, the statistical method employed, specifically mixed models, is known for its effectiveness in handling such unbalanced datasets. For example, these models assign greater weight to children with a higher number of measurements, thereby accommodating variations in data collection across participants. The inter-subject heterogeneity was high due to the differences in medical and clinical background such as clinical trial participation, corticosteroid intake, gene mutation, adherence to nighttime AFOs, periods of serial casting, functional level, etc. (S4 Table; S7-S9 Figs; S11-S14 Figs). The inter-subject variability was accounted for by using mixed models, enabling the delineation of the unique disease progression of each boy with DMD. However, the interpretation of the average trajectories was still influenced by these medical and clinical background differences and the sample size of the dataset was too small to test the impact of these effects. There is an increased need for large-scale multicenter studies with large sample sizes to delineate the influence of these background differences on the longitudinal trajectories. For the strength measurements, we were dependent on the cooperation and motivation of the children. Caution is needed with the interpretation of the unit-less ROMs trajectories, as they were calculated based on age categories, which could result in abrupt changes in the unit-less ROMs with age. This highlights the need for TD percentile curves for ROMs to aid in distinguishing pathological reductions in ROM from the typical reduction in ROM during growth. Lastly, the ultrasound dataset had a smaller sample size compared to the ROM and strength datasets due to the high processing burden. The selection of ultrasound data for processing was done independently of age and disease progression. However, in some older or more severely affected children, processing the m. rectus femoris data was not feasible due to muscle border invisibility caused by severe degeneration. As a result, the findings for the m. rectus femoris are not generalizable to older or more affected children. Additionally, due to the small sample size of the ultrasound dataset, these results should be interpreted with caution. Moreover, ultrasound has some inherent limitations compared to MRI. Ultrasound only measures superficial muscles, whereas MRI provides a comprehensive estimation of total muscle mass throughout the scanned limb. Additionally, ultrasound was used to measure CSA at 50% of the muscle belly length, which may not correspond to the maximum CSA of the muscle, potentially affecting our results. Another limitation is that we measured only one of the four quadriceps muscles involved in knee extension strength and one plantar flexor muscle involved in plantar flexion strength.

Despite the limitations, this study has established longitudinal trajectories of muscle impairments, consisting of muscle weakness, contractures, and muscle size alterations, in a cohort of growing boys with DMD covering the ambulation period. Moreover, the results provided enriched historical data and revealed promising outcome measures that could enhance the detection of the efficacy of novel therapeutic strategies. However, future large-scale multicenter studies with larger sample sizes are necessary to assess the impact of clinical and medical background differences among boys with DMD.

## Supporting information

S1 Appendix Standardized instrument strength assessment.
This document provides details on the measurement protocol of the instrumented strength assessment.
(DOCX)

S2 Appendix Standardized clinical examination using goniometry to measure passive joint range of motion.
This document provides details on the measurement protocol of the standardized clinical examination.
(DOCX)

S3 Appendix Standardized 3D freehand ultrasound.
This document provides details on the measurement protocol of the standardized 3D freehand ultrasound.
(DOCX)

S1 Table Fixed effects of linear mixed-effect models for the longitudinal trajectories of the muscle strength outcomes with age in boys with DMD. p-values in bold indicate significance level at p <  0.05. The following symbols represent: α_0_ =  intercept; β_1_ =  regression slope of age; β_2_ =  regression slope of age^2^; β_3_ =  regression slope of age^3^. CI, 95% confidence interval; DMD, Duchenne muscular dystrophy; n, number; obs, observations; subj, subjects.
(DOCX)

S2 Table Fixed effects of linear mixed-effect models for the longitudinal trajectories of the absolute and unit-less ROMs with age in boys with DMD. p-values in bold indicate significance level at p <  0.05. The following symbols represent: α_0_ =  intercept; β_1_ =  regression slope of age; β_2_ =  regression slope of age^2^; β_3_ =  regression slope of age^3^. CI, 95% confidence interval; DMD, Duchenne muscular dystrophy; n, number; obs, observations; ROM, range of motion; subj, subjects.
(DOCX)

S3 Table Fixed effects of linear mixed-effect models for the longitudinal trajectories of the muscle size alterations with age in boys with DMD. p-values in bold indicate significance level at p <  0.05. The following symbols represent: α_0_ =  intercept; β_1_ =  regression slope of age; β_2_ =  regression slope of age^2^. CI, 95% confidence interval; CSA, cross-sectional area; DMD, Duchenne muscular dystrophy; n, number; obs, observations; subj, subjects.
(DOCX)

S4 Table Clinical and medical background of the included patients.
(DOCX)

S5 Table Estimates of random-effect and residual covariance structure of piecewise models for the longitudinal trajectories of the muscle impairments with age in boys with DMD. The following symbols represent: σ^2^ = variance; a_i0_ =  random intercept; b_i_ =  random slope; b_i1_ =  random slope for regression slope before breakpoint; b_i2_ =  random slope for regression slope after breakpoint; ε_ij_ =  measurement error. CSA, cross-sectional area; DMD, Duchenne muscular dystrophy; ROM, range of motion.
(DOCX)

S6 Table Estimates of random-effect and residual covariance structure of linear mixed-effect models for the longitudinal trajectories of the muscle impairments with age in boys with DMD.
The following symbols represent: σ^2^ = variance; a_i0_ =  random intercept; b_i1_ =  random slope for regression slope of age; ε_ij_ =  measurement error. CSA, cross-sectional area; DMD, Duchenne muscular dystrophy; ROM, range of motion.
(DOCX)

S1 Fig Overview of the number of repeated assessments, time interval between assessments, and total follow-up time per participant with DMD, depicted across the age range.
**Every patient is represented in a different color. (A) Total database. (B) Strength dataset. (C) Total database, with measurements collected retrospectively shown in light gray and prospectively in dark gray. (D) ROM dataset. (E) Ultrasound dataset.** DMD, Duchenne muscular dystrophy; ROM, range of motion.
(TIF)

S2 Fig Absolute strength outcomes (i.e., joint moments) of the participants with DMD projected on anthropometric-related TD percentile curves for (A) hip, (B) knee, and (C) ankle muscle strength.
Estimated percentiles 5% (P5), 10% (P10), 25% (P25), 50% (P50), 75% (P75), 90% (P90), 95% (P95) of the change in absolute joint moments with increasing anthropometric values (i.e., body mass x height) of TD children are plotted. The colored dots indicate the absolute joint moments of the participants with DMD, with each participant represented in a different color. These curves were used to convert the absolute joint moments of the participants with DMD into unit-less z-scores, where P5 corresponds to a z-score of −1.645, P10 to −1.282, P25 to −0.675, P50 to 0, P75 to 0.675, P90 to 1.282, and P95 to 1.645. A z-score of 0 means that the absolute joint moment equals the median absolute joint moment of TD children. A z-score of +/−1 indicates that the absolute joint moment deviates by one standard deviation above or below the median of TD children, respectively. DMD, Duchenne muscular dystrophy; TD, typically developing.
(TIF)

S3 Fig Absolute muscle size outcomes (i.e., cross-sectional area) of the participants with DMD projected on anthropometric-related TD percentile curves for muscle size.
Estimated percentiles 5% (P5), 10% (P10), 25% (P25), 50% (P50), 75% (P75), 90% (P90), 95% (P95) of the change in absolute cross-sectional area with increasing body mass of TD children are plotted. The colored dots indicate the absolute cross-sectional areas of the participants with DMD, with each participant represented in a different color. These curves were used to convert the absolute cross-sectional areas of the participants with DMD into unit-less z-scores, where P5 corresponds to a z-score of -1.645, P10 to -1.282, P25 to -0.675, P50 to 0, P75 to 0.675, P90 to 1.282, and P95 to 1.645. A z-score of 0 means that the absolute cross-sectional area equals the median absolute cross-sectional area of TD children. A z-score of +/−1 indicates that the absolute cross-sectional area deviates by one standard deviation above or below the median of TD children, respectively. DMD, Duchenne muscular dystrophy; TD, typically developing.
(TIF)

S4 Fig Predicted longitudinal trajectories for strength of (A) hip, (B) knee, and (C) ankle muscles with age in boys with DMD of linear mixed-effect models.
The average predicted trajectory (thick black line), the individual predicted profiles (thinner colored lines), and the actual observed outcomes (colored dots) are displayed. Each color represents one patient with DMD. The estimates for the fixed effects are given in S1 Table. DMD, Duchenne muscular dystrophy.
(TIF)

S5 Fig Predicted longitudinal trajectories for (left column) absolute and (right column) unit-less (A) knee extension ROM, (B) hamstrings ROM, (C) ankle dorsiflexion with knee extended, and (D) ankle dorsiflexion with knee flexed with age in boys with DMD of linear mixed-effect models.
The average predicted trajectory (thick black line), the individual predicted profiles (thinner colored lines), and the actual observed outcomes (colored dots) are displayed. Each color represents one patient with DMD. The estimates for the fixed effects are given in S2 Table. DMD, Duchenne muscular dystrophy; ROM, range of motion.
(TIF)

S6 Fig Predicted longitudinal trajectories for muscle size alterations with age in boys with DMD of linear mixed-effect models.
The average predicted trajectory (thick black line), the individual predicted profiles (thinner colored lines), and the actual observed outcomes (colored dots) are displayed. Each color represents one patient with DMD. The estimates for the fixed effects are given in S3 Table. DMD, Duchenne muscular dystrophy.
(TIF)

S7 Fig Overview of the clinical and medical background of the included participants.
**Each color represents one patient with DMD.** AFO, ankle foot orthosis; DMD, Duchenne muscular dystrophy; LA, loss of ambulation.
(TIF)

S8 Fig Data exploration of the impact of clinical trial participation with disease-modifying medication on the individual predicted trajectories for strength of (A) hip, (B) knee, and (C) ankle muscles with age.
The observed actual values during clinical trial participation are color-coded: red for Ataluren, orange for Exon-skipping 45, yellow for Exon-skipping 51, green for Exon-skipping 53, blue for Givinostat, navy blue for Tadalafil, and purple for Vamorolone. If a boy participated in a clinical trial at any point during follow-up, his entire predicted profile is displayed in the corresponding color. For boys who did not participate in any clinical trials, both the observed values and predicted profiles are shown in gray. No major conclusions can be drawn from this data exploration due to significant variability among children within the same trials.
(TIF)

S9 Fig Visual comparison of the individual predicted trajectories for strength of (A) hip, (B) knee, and (C) ankle muscles with age, between boys who lost ambulation within 1 year after the last measurement (in green) and boys who continued walking for more than 1 year after the last measurement (in gray).
Boys who lost ambulation overall showed lower z-scores, especially for hip and knee extension strength.
(TIF)

S10 Fig Data exploration of longitudinal trajectories for (left column) absolute and (right column) unit-less (A) hip extension ROM, and (B) hip adduction ROM with age in boys with DMD of linear mixed-effect models.
The Loess regression (thick black line) and the actual observed outcomes (colored symbols) are displayed. Each color represents one patient with DMD. DMD, Duchenne muscular dystrophy; ROM, range of motion.
(TIF)

S11 Fig Data exploration of the impact of clinical trial participation with disease-modifying medication on the individual predicted trajectories for (left column) absolute and (right column) unit-less (A) knee extension ROM, (B) hamstrings ROM, (C) ankle dorsiflexion with knee extended, and (D) ankle dorsiflexion with knee flexed with age.
The observed actual values during clinical trial participation are color-coded: red for Ataluren, orange for Exon-skipping 45, yellow for Exon-skipping 51, green for Exon-skipping 53, blue for Givinostat, navy blue for Tadalafil, and purple for Vamorolone. If a boy participated in a clinical trial at any point during follow-up, his entire predicted profile is displayed in the corresponding color. For boys who did not participate in any clinical trials, both the observed values and predicted profiles are shown in gray. No major conclusions can be drawn from this data exploration due to significant variability among children within the same trials. ROM, range of motion.
(TIF)

S12 Fig Visual comparison of the individual predicted trajectories for (left column) absolute and (right column) unit-less (A) knee extension ROM, (B) hamstrings ROM, (C) ankle dorsiflexion with knee extended, and (D) ankle dorsiflexion with knee flexed with age, between boys who lost ambulation within 1 year after the last measurement (in green) and boys who continued walking for more than 1 year after the last measurement (in gray).
Boys who lost ambulation overall showed lower z-scores, especially for knee extension ROM. ROM, range of motion.
(TIF)

S13 Fig Data exploration of the impact of clinical trial participation with disease-modifying medication on the individual predicted trajectories for muscle size alterations with age.
The observed actual values during clinical trial participation are color-coded: red for Ataluren, orange for Exon-skipping 45, yellow for Exon-skipping 51, green for Exon-skipping 53, blue for Givinostat, navy blue for Tadalafil, and purple for Vamorolone. If a boy participated in a clinical trial at any point during follow-up, his entire predicted profile is displayed in the corresponding color. For boys who did not participate in any clinical trials, both the observed values and predicted profiles are shown in gray. No major conclusions can be drawn from this data exploration due to significant variability among children within the same trials.
(TIF)

S14 Fig Visual comparison of the individual predicted trajectories for muscle size alterations with age, between boys who lost ambulation within 1 year after the last measurement (in green) and boys who continued walking for more than 1 year after the last measurement (in gray).
No major conclusion can be drawn. It was often not feasible to process the data for the m. rectus femoris in more severely affected children, resulting in data exclusion and making the dataset not fully generalizable.
(TIF)
