# Supplementary material for: Longitudinal trajectories of muscle impairments in growing boys with Duchenne muscular dystrophy
Source: PLoS One. 2025 Mar 18;20(3):e0307007. doi: 10.1371/journal.pone.0307007 (PMC11918350; doi:10.1371/journal.pone.0307007)
Supplement: S1 Appendix — This document provides details on the measurement protocol of the instrumented strength assessment. (DOCX) [file pone.0307007.s001.docx]

**S1 Appendix: Standardized instrumented strength assessment**


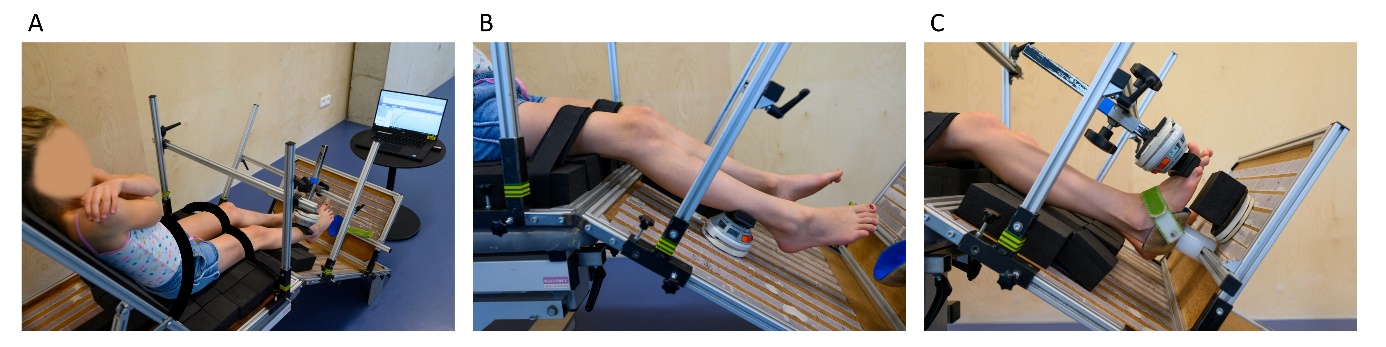


Muscle strength was assessed by performing maximal isometric voluntary contractions using a fixed dynamometer (MicroFet, Hogan Health Industries, West Jordan, UT United States) in a standardized test position [1,2]. Hereto, both participant and dynamometer were fixated on a custom-made chair to decrease compensations and to eliminate the influence of the assessor strength [1]. The standardized test position was 60° of hip flexion, 30° of knee flexion and the ankle joint in neutral position. The dynamometer was placed at 75% of the upper leg length (i.e., distance between trochanter major and knee joint space) to assess the strength of the hip muscles, at 75% of the lower leg length (i.e., distance between proximal border of fibula head and distal border of lateral malleolus) to assess the strength of the knee muscles, and at 75% of the foot length (i.e., distance between distal border (dorsal point) of the lateral malleolus and the distal metatarsal II, projected on the lateral border of the foot) to assess the strength of the ankle muscles. During the entire assessment, the pelvis was fixated with a strap and the participants crossed their arms in front of their chest. The upper legs of the participants were fixated with straps during the assessments of the knee and ankle muscles (not the hip muscles). The heel was fixated in a heel cuff for the assessment of hip extension, hip flexion, plantar flexion and dorsiflexion strength. To account for the influence of gravitational force during the assessment of hip extension, knee flexion and plantar flexion strength, a separate passive trial was conducted and subtracted afterwards from the outcomes of the maximal voluntary isometric contractions [3]. Each assessment consisted of a test trial, followed by three well executed trials with a duration of 3-5s. If compensations were noticed, such as visible contractions in muscles other than the one being tested, an extra trial was conducted after giving verbal instructions to ensure proper execution and to minimize compensations. A minimum rest period of 10 seconds was provided between trials. If signs of fatigue were detected, the recovery period was extended until the participant was prepared to continue. When moving on to measurements of a different joint, a rest period of at least two minutes was provided. Throughout the assessment, the children received steady verbal encouragement from the assessor, along with visual feedback of both the current and previous trials, which is shown in the background on the figure in panel A. Panel B provides a close-up of the positioning for the knee flexion strength assessment, while Panel C shows the positioning for the dorsiflexion strength assessment.

References:

1. Goudriaan M, Nieuwenhuys A, Schless S, Goemans N, Molenaers G, Desloovere K. A new strength assessment to evaluate the association between muscle weakness and gait pathology in children with cerebral palsy. PLoS One. 2018;13: e0191097. doi:10.1371/journal.pone.0191097

2. Verreydt I, Vandekerckhove I, Stoop E, Peeters N, van Tittelboom V, Van de Walle P, et al. Instrumented strength assessment in typically developing children and children with a neural or neuromuscular disorder: A reliability, validity and responsiveness study. Front Physiol. 2022;13: 855222. doi:10.3389/fphys.2022.855222

3. Boiteau M, Malouin F, Richards CL. Use of a hand-held dynamometer and a Kin-Com® dynamometer for evaluating spastic hypertonia in children: A reliability study. Phys Ther. 1995;75: 796–802. doi:10.1093/ptj/75.9.796
