# Supplementary material for: Longitudinal trajectories of muscle impairments in growing boys with Duchenne muscular dystrophy
Source: PLoS One. 2025 Mar 18;20(3):e0307007. doi: 10.1371/journal.pone.0307007 (PMC11918350; doi:10.1371/journal.pone.0307007)
Supplement: S2 Appendix — This document provides details on the measurement protocol of the standardized clinical examination. (DOCX) [file pone.0307007.s002.docx]

| **Test** | **Position** | **Passive movement** | **Measurement of angle** |
| --- | --- | --- | --- |
| Modified Thomas test [1] | Supine with the pelvis located at the end of the table | The assessor flexes both legs to the chest to ensure a standardized position of the pelvis (ASIS aligned over the PSIS), then the assessor keeps the contralateral leg in this position while passively moving the ipsilateral leg to full hip extension. A stretch is provided at the end of the movement to ensure measuring the end ROM. The ASIS is palpated throughout the passive movement to control for movement of the pelvis. | The angle between the horizontal axis and the long axis of the femur is measured with a goniometer. Hip extension beyond neutral is recorded as a positive angle. Hip flexion is recorded as a negative angle. |
| Hip adduction [2] | Supine, anatomical position | An assessor flexes the contralateral leg in 90° hip and knee flexion. A second assessor passively moves the ipsilateral leg towards hip adduction. A stretch is provided at the end of the movement to ensure measuring the end ROM. The ASIS is palpated throughout the passive movement to control for movement of the pelvis | The angle between the extended line of the trunk (or the long axis of the femur in anatomical position) and the long axis of the femur is measured with a goniometer. Hip adduction beyond neutral is recorded as a positive angle. Hip abduction is recorded as a negative angle. |
| Knee extension [1] | Supine, anatomical position | The assessor passively extends the knee by pulling the tibia up and pushing the femur down. A stretch is provided at the end of the movement to ensure measuring the end ROM. | The angle between the long axis of the femur and the long axis of the tibia is measured with a goniometer. Knee hyperextension beyond neutral is recorded as a positive angle. Knee flexion is recorded as a negative angle. |
| True popliteal angle [1] | Supine, hip flexion in both legs | The assessor flexes both legs to ensure a standardized position of the pelvis (ASIS aligned over the PSIS), then the assessor passively extends the knee towards knee extension, while the hip is in 90° of hip flexion. A stretch is provided at the end of the movement to ensure measuring the end ROM. The ASIS is palpated throughout the passive movement to control for movement of the pelvis | The angle between the vertical and the long axis of the femur is measured with a goniometer. The angle is recorded as a negative angle. |
| Ankle dorsiflexion with knee extended [1] | Supine, anatomical position | The assessor passively moves the ankle towards dorsiflexion, while keeping the knee in full extension. The movement of the assessor is initiated at the heel (fingers wrap around the heel) to avoid increased motion throughout the midfoot. A stretch is provided at the end of the movement to ensure measuring the end ROM. | The angle from the anatomical position. Dorsiflexion beyond the anatomical position is recorded as a positive angle. Plantar flexion is recorded as a negative angle. |
| Ankle dorsiflexion with knee flexed [1] | Supine, ipsilateral leg is in 90° knee flexion | The assessor passively moves the ankle towards dorsiflexion, while keeping the knee in 90° of flexion. The movement of the assessor is initiated at the heel (fingers wrap around the heel) to avoid increased motion throughout the midfoot. A stretch is provided at the end of the movement to ensure measuring the end ROM. | The angle from the anatomical position. Dorsiflexion beyond the anatomical position is recorded as a positive angle. Plantar flexion is recorded as a negative angle. |

**S2 Appendix:** **Standardized clinical examination using goniometry to measure passive joint range of motion**

Abbreviations: ASIS, anterior superior iliac spine; PSIS, posterior superior iliac spine; ROM, range of motion.

References:

1. Mudge AJ, Bau K V., Purcell LN, Wu JC, Axt MW, Selber P, et al. Normative reference values for lower limb joint range, bone torsion, and alignment in children aged 4-16 years. J Pediatr Orthop Part B. 2014;23: 15–25. doi:10.1097/BPB.0b013e328364220a

2. Sankar WN, Laird CT, Baldwin KD. Hip range of motion in children: What is the norm? J Pediatr Orthop. 2012;32: 399–405. doi:10.1097/BPO.0b013e3182519683
