# Supplementary material for: Longitudinal trajectories of muscle impairments in growing boys with Duchenne muscular dystrophy
Source: PLoS One. 2025 Mar 18;20(3):e0307007. doi: 10.1371/journal.pone.0307007 (PMC11918350; doi:10.1371/journal.pone.0307007)
Supplement: S3 Appendix — This document provides details on the measurement protocol of the standardized 3D freehand ultrasound. (DOCX) [file pone.0307007.s003.docx]

**S3 Appendix: Standardized 3D freehand ultrasound** [1–4]


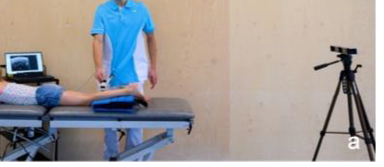

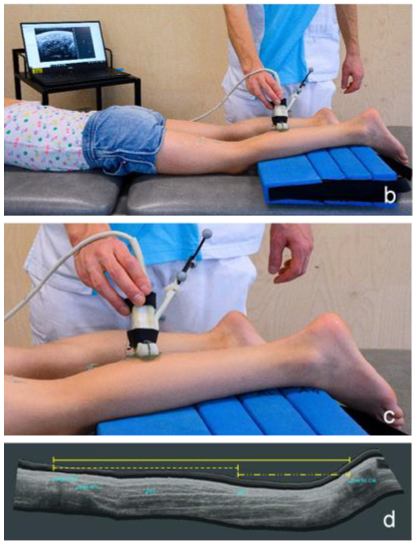

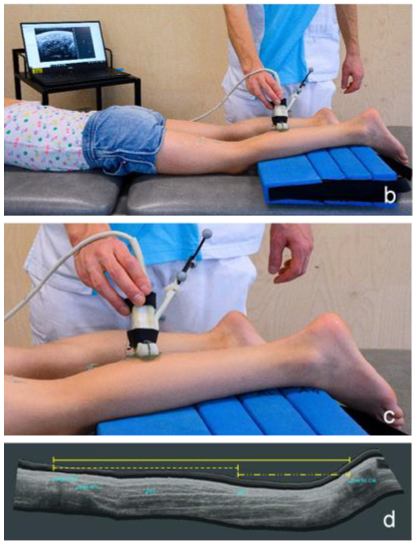

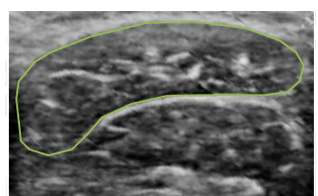


e

Muscle size of the m. rectus femoris, m. tibialis anterior and m. medial gastrocnemius was measured using 3D freehand ultrasound. Ultrasound images were captured by a conventional two-dimensional ultrasound machine (HL9.0/60/128Z, Telemed EchoBlaster 128 Ext-1Z system, Lithuania) using a linear transducer (59 mm field of view). This setup was integrated with a motion tracking system (featuring 3 cameras and 1 mm resolution, Optitrack NaturalPoint, USA) (a). The motion tracking system recorded the position and orientation of four reflective markers, which were attached to the ultrasound transducer (b-c). The ultrasound signal and motion-tracking signal were synchronized. To minimize muscle deformation during the acquisitions, the Portico (i.e., a custom shaped gel pad placed in a concave plastic mount) was used with sufficient amounts of acoustic transmission gel (c) [2].

During the acquisition, participants lay relaxed on an examination table (b). The m. rectus femoris and m. tibialis anterior were assessed in supine and the m. medial gastrocnemius was assessed in prone. A triangular cushion was positioned under the lower leg, resulting in approximately 25° of knee flexion and an unconstrained ankle position (approximately 30° on average) (b). The hip was flexed approximately 20° during the measurement of the m. rectus femoris.

During the acquisition, the US probe was held perpendicular to the deep aponeurosis of the muscle, whilst sweeping over the targeted muscle at a constant velocity (30 images per second) to acquire US images in a transverse orientation (c). Muscles were scanned from proximal to distal.

Throughout the procedure, the child was instructed to remain as still as possible. If muscle contraction or movement occurred, the acquisition was repeated. Acquisition parameters, including frequency (10 MHz), focus (1.8–2.8 cm), gain (46%), dynamic range (44 dB), and time-gain compensation settings, were kept consistent. The default image depth was set to 5 cm but could be adjusted to 7 cm to capture the deeper muscle borders of larger muscles like the m. medial gastrocnemius and m. rectus femoris in older children.

In general, a single sweep was sufficient for acquisition. However, if the muscle width exceeded the transducer’s width, multiple parallel sweeps were taken and combined during processing. Data collection and synchronization were managed with STRADWIN software (version 6.0; Mechanical Engineering, Cambridge University).

Post-processing of the dataset in STRADWIN (d) involved defining muscle belly length by marking relevant anatomical landmarks (proximal landmarks: m. rectus femoris origin at spina iliaca anterior inferior; m. tibialis anterior origin at the fronto-lateral surface of the tibia; m. medial gastrocnemius origin at the medial femoral condyle; distal landmarks: muscle-tendon junctions) and calculating the linear distance between these landmarks (d). The mid-belly cross-sectional area (in mm²) was then determined by drawing a segmentation along the inside of the muscle border at 50% of the muscle belly length (e).

In cases where muscle degeneration significantly impaired the visibility of the muscle border, a single 2D image was captured at the mid-belly during acquisition. Real-time feedback from ultrasound images, combined with palpation, was used to identify landmarks superficially on the leg’s exterior. A tape measure was then used to determine the midpoint between these landmarks (i.e., mid-belly) on the leg's exterior.

References:

1. Cenni F, Schless SH, Bar-On L, Aertbeliën E, Bruyninckx H, Hanssen B, et al. Reliability of a clinical 3D freehand ultrasound technique: Analyses on healthy and pathological muscles. Comput Methods Programs Biomed. 2018;156: 97–103. doi:10.1016/j.cmpb.2017.12.023

2. Cenni F, Schless SH, Monari D, Bar-On L, Aertbeliën E, Bruyninckx H, et al. An innovative solution to reduce muscle deformation during ultrasonography data collection. J Biomech. 2018;77: 194–200. doi:10.1016/j.jbiomech.2018.06.002

3. Hanssen B, Peeters N, Dewit T, Huyghe E, Dan B, Molenaers G, et al. Reliability of 3D freehand ultrasound to assess lower limb muscles in children with spastic cerebral palsy and typical development. J Anat. 2023;242: 986–1002. doi:10.1111/joa.13839

4. Peeters N, Hanssen B, De Beukelaer N, Vandekerckhove I, Walhain F, Huyghe E, et al. A comprehensive normative reference database of muscle morphology in typically developing children aged 3–18 years—a cross-sectional ultrasound study. J Anat. 2023;242: 754–770. doi:10.1111/joa.13817
