# Supplementary material for: Longitudinal trajectories of muscle impairments in growing boys with Duchenne muscular dystrophy
Source: PLoS One. 2025 Mar 18;20(3):e0307007. doi: 10.1371/journal.pone.0307007 (PMC11918350; doi:10.1371/journal.pone.0307007)
Supplement: S1 Table — The following symbols represent: α0 = intercept; β1 = regression slope of age; β2 = regression slope of age2; β3 = regression slope of age3. CI, 95% confidence interval; DMD, Duchenne muscular dystrophy; n, number; obs, observations; subj, subjects. (DOCX) [file pone.0307007.s004.docx]

**S1 Table. Fixed effects of linear mixed-effect models for the longitudinal trajectories of the muscle strength deficits with age for boys with DMD**

|  |  |  | **Intercept** |  | **Regression coefficients (β)** | | | | |
| --- | --- | --- | --- | --- | --- | --- | --- | --- | --- |
|  |  |  | α_0_ (CI) |  | β_1_ (CI) |  | β_2_ (CI) |  | β_3_ (CI) |
| **Outcomes** | n subj | n obs | *p-value* |  | *p-value* |  | *p-value* |  | *p-value* |
| Hip extension strength  (z-score) | 31 | 141 | -11.77 (-19.71 -3.82) |  | 3.04 (0.65 5.44) |  | -0.31 (-0.53 -0.09) |  | 0.009 (0.003 0.016) |
|  |  |  | ***0.0050*** |  | ***0.0147*** |  | ***0.0067*** |  | ***0.0076*** |
| Hip flexion strength  (z-score) | 31 | 141 | -11.77 (-20.39 -3.15) |  | 3.44 (0.78 6.10) |  | -0.34 (-0.62 -0.07) |  | 0.010 (0.001 0.019) |
|  |  |  | ***0.0091*** |  | ***0.0119*** |  | ***0.0150*** |  | ***0.0270*** |
| Hip abduction strength  (z-score) | 31 | 140 | -12.23 (-17.77 -6.70) |  | 3.43 (1.70 5.15) |  | -0.34 (-0.51 -0.16) |  | 0.010 (0.004 0.015) |
|  |  |  | ***<0.0001*** |  | ***0.0001*** |  | ***0.0002*** |  | ***0.0008*** |
| Knee extension strength  (z-score) | 31 | 161 | -5.30 (-9.40 -1.20) |  | 1.80 (0.43 3.18) |  | -0.20 (-0.34 -0.06) |  | 0.006 (0.001 0.011) |
|  |  |  | ***0.0130*** |  | ***0.0122*** |  | ***0.0057*** |  | ***0.0153*** |
| Knee flexion strength  (z-score) | 31 | 161 | -0.26 (-1.20 0.67) |  | -0.15 (-0.24 -0.07) |  |  |  |  |
|  |  |  | *0.5704* |  | ***0.0011*** |  |  |  |  |
| Plantar flexion strength  (z-score) | 31 | 161 | -4.68 (-7.86 -1.50) |  | 0.81 (0.16 1.46) |  | -0.05 (-0.08 -0.02) |  |  |
|  |  |  | ***0.0053*** |  | ***0.0160*** |  | ***0.0037*** |  |  |
| Dorsiflexion strength  (z-score) | 31 | 161 | -3.22 (-5.76 -0.68) |  | 0.39 (-0.09 0.88) |  | -0.03 (-0.05 -0.01) |  |  |
|  |  |  | ***0.0147*** |  | *0.1082* |  | ***0.0044*** |  |  |

p-values in bold indicate significance level at p < 0.05.

The following symbols represent: α_0_ = intercept; β_1_ = regression slope of age; β_2_ = regression slope of age^2^; β_3_ = regression slope of age^3^.

CI, 95% confidence interval; DMD, Duchenne muscular dystrophy; n, number; obs, observations; subj, subjects
