# Supplementary material for: Longitudinal trajectories of muscle impairments in growing boys with Duchenne muscular dystrophy
Source: PLoS One. 2025 Mar 18;20(3):e0307007. doi: 10.1371/journal.pone.0307007 (PMC11918350; doi:10.1371/journal.pone.0307007)
Supplement: S2 Table — The following symbols represent: α0 = intercept; β1 = regression slope of age; β2 = regression slope of age2; β3 = regression slope of age3. CI, 95% confidence interval; DMD, Duchenne muscular dystrophy; n, number; obs, observations; ROM, range of motion; subj, subjects. (DOCX) [file pone.0307007.s005.docx]

**S2 Table. Fixed effects of linear mixed-effect models for the longitudinal trajectories of the absolute and unit-less ROMs with age for boys with DMD**

|  |  |  | **Intercept** |  | **Regression coefficients (β)** | | | | |
| --- | --- | --- | --- | --- | --- | --- | --- | --- | --- |
|  |  |  | α_0_ (CI) |  | β_1_ (CI) |  | β_2_ (CI) |  | β_3_ (CI) |
| **Outcomes** | n subj | n obs | *p-value* |  | *p-value* |  | *p-value* |  | *p-value* |
| Knee extension ROM (°) | 32 | 176 | -14.09 (-27.78 -0.40) |  | 6.11 (1.55 10.68) |  | -0.59 (-1.07 -0.11) |  | 0.017 (0.001 0.033) |
|  |  |  | ***0.0441*** |  | ***0.0103*** |  | ***0.0164*** |  | ***0.0341*** |
| Knee extension ROM (z-score) | 32 | 176 | -0.27 (-1.03 0.49) |  | 0.06 (-0.03 0.15) |  |  |  |  |
|  |  |  | *0.4775* |  | *0.1808* |  |  |  |  |
| Hamstrings ROM  (°) | 32 | 176 | -40.47 (-50.12 -30.83) |  | 0.42 (-0.42 1.26) |  |  |  |  |
|  |  |  | ***<0.0001*** |  | *0.3263* |  |  |  |  |
| Hamstrings ROM (z-score) | 32 | 176 | -2.28 (-3.21 -1.35) |  | 0.10 (0.01 0.19) |  |  |  |  |
|  |  |  | ***<0.0001*** |  | ***0.0239*** |  |  |  |  |
| Dorsiflexion ROM  knee extended (°) | 32 | 178 | -20.09 (-43.25 3.07) |  | 10.51 (3.33 17.69) |  | -1.10 (-1.81 -0.39) |  | 0.030 (0.008 0.052) |
|  |  |  | *0.0866* |  | ***0.0044*** |  | ***0.0025*** |  | ***0.0070*** |
| Dorsiflexion ROM  knee extended (z-score) | 32 | 178 | -2.95 (-4.88 -1.01) |  | 0.23 (-0.15 0.60) |  | -0.02 (-0.04 -0.01) |  |  |
|  |  |  | ***0.0040*** |  | *0.2346* |  | ***0.0131*** |  |  |
| Dorsiflexion ROM  knee flexed (°) | 32 | 178 | 27.12 (21.33 32.91) |  | -1.73 (-2.19 -1.28) |  |  |  |  |
|  |  |  | ***<0.0001*** |  | ***<0.0001*** |  |  |  |  |
| Dorsiflexion ROM  knee flexed (z-score) | 32 | 178 | -0.84 (-1.69 0.01) |  | -0.17 (-0.24 -0.11) |  |  |  |  |
|  |  |  | *0.0532* |  | ***<0.0001*** |  |  |  |  |

p-values in bold indicate significance level at p < 0.05.

The following symbols represent: α_0_ = intercept; β_1_ = regression slope of age; β_2_ = regression slope of age^2^; β_3_ = regression slope of age^3^.

CI, 95% confidence interval; DMD, Duchenne muscular dystrophy; n, number; obs, observations; ROM, range of motion; subj, subjects
