# Supplementary material for: Longitudinal trajectories of muscle impairments in growing boys with Duchenne muscular dystrophy
Source: PLoS One. 2025 Mar 18;20(3):e0307007. doi: 10.1371/journal.pone.0307007 (PMC11918350; doi:10.1371/journal.pone.0307007)
Supplement: S3 Table — The following symbols represent: α0 = intercept; β1 = regression slope of age; β2 = regression slope of age2. CI, 95% confidence interval; CSA, cross-sectional area; DMD, Duchenne muscular dystrophy; n, number; obs, observations; subj, subjects. (DOCX) [file pone.0307007.s006.docx]

**S3 Table. Fixed effects of linear mixed-effect models for the longitudinal trajectories of the muscle size alterations with age for boys with DMD**

|  |  |  | **Intercept** |  | **Regression coefficients (β)** | | | | |
| --- | --- | --- | --- | --- | --- | --- | --- | --- | --- |
|  |  |  | α_0_ (CI) |  | β_1_ (CI) |  | β_2_ (CI) |  | β_3_ (CI) |
| **Outcomes** | n subj | n obs | *p-value* |  | *p-value* |  | *p-value* |  | *p-value* |
| Rectus femoris  CSA (z-score) | 23 | 44 | -6.31 (-11.46 -1.17) |  | 2.36 (0.55 4.16) |  | -0.23 (-0.42 -0.05) |  | 0.007 (0.001 0.013) |
|  |  |  | ***0.0185*** |  | ***0.0133*** |  | ***0.0172*** |  | ***0.0279*** |
| Medial gastrocnemius  CSA (z-score) | 32 | 64 | -8.80 (-18.92 1.33) |  | 4.08 (0.79 7.37) |  | -0.40 (-0.73 -0.06) |  | 0.011 (0.001 0.022) |
|  |  |  | *0.0862* |  | ***0.0169*** |  | ***0.0210*** |  | ***0.0391*** |
| Tibialis anterior  CSA (z-score) | 28 | 62 | -2.36 (-5.78 1.06) |  | 0.72 (0.09 1.36) |  | -0.035 (-0.067 -0.004) |  |  |
|  |  |  | *0.1679* |  | ***0.0281*** |  | ***0.0304*** |  |  |

p-values in bold indicate significance level at p < 0.05.

The following symbols represent: α_0_ = intercept; β_1_ = regression slope of age; β_2_ = regression slope of age^2^, β_3_ = regression slope of age^3^.

CI, 95% confidence interval; CSA, cross-sectional area; DMD, Duchenne muscular dystrophy; n, number; obs, observations; subj, subjects
