# Supplementary material for: Longitudinal trajectories of muscle impairments in growing boys with Duchenne muscular dystrophy
Source: PLoS One. 2025 Mar 18;20(3):e0307007. doi: 10.1371/journal.pone.0307007 (PMC11918350; doi:10.1371/journal.pone.0307007)
Supplement: S4 Table — (DOCX) [file pone.0307007.s007.docx]

| **Patients** | **Number of repeated assessments** | **Time interval (in years; min-max)** | **Follow-up time (in years)** | **Mutation type** | **Exon** | **Corticosteroids** | **Dose Corticosteroids** | **Clinical trials** |
| --- | --- | --- | --- | --- | --- | --- | --- | --- |
| 1 | 3 | 1.0-1.1 | 2.1 | Deletion | 51 | Vamorolone | 2.2 ml | VBP15-004/VISION DMD |
| 2 | 3 | 1.0-1.0 | 2 | Deletion | 45 | Vamorolone | 1.8 ml | VBP15-004/VISION DMD |
| 3 | 6 | 0.5-1.0 | 4.1 | Deletion | 8 | Deflazacort | 15 mg; 21 mg |  |
| 4 | 2 | 1.3 | 1.3 | Deletion | 45 | Deflazacort | 18 mg |  |
| 5 | 5 | 0.5-1.1 | 3 | Deletion | 24 | Deflazacort | 12 mg; 15 mg |  |
| 6 | 6 | 0.5-1.0 | 4 | Deletion | 46-52 | Deflazacort | 15 mg |  |
| 7 | 1 | 0 | 0 | Deletion | 45-50 | Deflazacort | 12 mg |  |
| 8 | 3 | 1.0-1.5 | 2.5 | Deletion | 45-50 | Deflazacort | 18 mg | WVE-DMDX51-003: 51 exon skipping; WVE-210201 (Sudovirsen) |
| 9 | 11 | 0.5-1.0 | 6 | Deletion | 50 | Deflazacort | 15 mg | WVE-DMDX51-001: 51 exon skipping; WVE-210201 (Sudovirsen) |
| 10 | 8 | 0.5-1.4 | 5.9 | Duplication | 74 | Deflazacort | 12 mg; 15 mg |  |
| 11 | 5 | 0.5-2.1 | 4.6 | Deletion | 45-52 | Deflazacort | 12 mg; 15 mg; 18 mg | Sarepta-Essence: SRP4045-SRP4053-301 study; exon skipping 53 |
| 12 | 4 | 0.6-2.3 | 3.8 | Deletion | 51-55 | Deflazacort | 18 mg |  |
| 13 | 6 | 0.5-1.1 | 4 | Duplication | 8-9 | Deflazacort | 18 mg |  |
| 14 | 11 | 0.5-1.0 | 6.2 | Deletion | 17 | Deflazacort | 12 mg; 15 mg |  |
| 15 | 11 | 0.5-1.0 | 6.2 | Deletion | 17 | Deflazacort | 12 mg; 15 mg |  |
| 16 | 8 | 0.5-0.5 | 3.6 | Deletion | 50-54 | Deflazacort | 15 mg; 18 mg | Italfarmaco-Givinostat: DSC/14/2357/48-51 (anti-fibroticum) |
| 17 | 2 | 2 | 2 | Deletion | 45-50 | Deflazacort | 15 mg | WVE-DMDX51-001: 51 exon skipping; WVE-210201 (Sudovirsen) |
| 18 | 2 | 0.6 | 0.6 | Deletion | 8-41 | Deflazacort | 21 mg |  |
| 19 | 11 | 0.5-1.9 | 6.4 | Duplication | 40 | Deflazacort | 18 mg | Italfarmaco-Givinostat: DSC/14/2357/48-51 (anti-fibroticum) |
| 20 | 4 | 0.5-1.6 | 3.2 | Deletion | 44 | Deflazacort | 15 mg; 18 mg | Sarepta-Essence: SRP4045-SRP4053-301 study: exon skipping 45 |
| 21 | 6 | 0.4-2.9 | 4.9 | Deletion | 16-17 | Deflazacort | 18 mg; 21 mg |  |
| 22 | 10 | 0.5-1.6 | 6.6 | Nonsense Point | 44 | Deflazacort | 18 mg; 21 mg | Ataluren PTC124-GD-020e-DMD (PTC-124) |
| 23 | 6 | 0.5-0.7 | 2.6 | Deletion | 1-49 | Deflazacort | 21 mg | Italfarmaco-Givinostat: DSC/14/2357/48-51 (anti-fibroticum) |
| 24 | 4 | 0.5-1.8 | 3.3 | Deletion | 17 | Deflazacort | 21 mg |  |
| 25 | 2 | 1 | 1 | Deletion | 46-49 | Deflazacort | 15 mg | Italfarmaco-Givinostat: DSC/14/2357/48-51 (anti-fibroticum) |
| 26 | 9 | 0.5-0.5 | 4 | Nonsense Point | 22 | Deflazacort | 21 mg; 24 mg |  |
| 27 | 4 | 0.5-1.8 | 2.8 | Deletion | 45-52 | Deflazacort | 18 mg |  |
| 28 | 4 | 0.5-1.6 | 3.2 | Deletion | 44 | Deflazacort | 18 mg | Sarepta-Essence: SRP4045-SRP4053-301 study: exon skipping 45 |
| 29 | 5 | 0.5-1.1 | 3.6 | Deletion | 46-49 | Deflazacort | 21 mg | Sarepta-Essence: SRP4045-SRP4053-301 study: exon skipping 45 |
| 30 | 8 | 0.4-0.6 | 3.5 | Nonsense Point | 41 | Deflazacort | 18 mg | Ataluren PTC124-GD-020e-DMD (PTC-124) |
| 31 | 5 | 0.5-2.5 | 4.5 | Duplication | 2 | Deflazacort | 18 mg | H6D-MC-LVJJ study (Tadalafil) & Italfarmaco-Givinostat: DSC/14/2357/48-51 (anti-fibroticum) |
| 32 | 2 | 0.5 | 0.5 | Duplication | 3-7 | Deflazacort | 21 mg |  |
| 33 | 4 | 0.4-1.0 | 2 | Deletion | 45-54 | Deflazacort | 12 mg |  |

**S4 Table:** **Clinical and medical background of the included patients**
