# Supplementary material for: Longitudinal trajectories of muscle impairments in growing boys with Duchenne muscular dystrophy
Source: PLoS One. 2025 Mar 18;20(3):e0307007. doi: 10.1371/journal.pone.0307007 (PMC11918350; doi:10.1371/journal.pone.0307007)
Supplement: S5 Table — The following symbols represent: σ2 = variance; ai0 = random intercept; bi = random slope; bi1 = random slope for regression slope before breakpoint; bi2 = random slope for regression slope after breakpoint; εij = measurement error. CSA, cross-sectional area; DMD, Duchenne muscular dystrophy; ROM, range of motion. (DOCX) [file pone.0307007.s008.docx]

**S5 Table. Estimates of random-effect and residual covariance structure of piecewise models for the longitudinal trajectories of the muscle impairments with age for boys with DMD**

|  |  | **Random effects** | |  | **Residual** |
| --- | --- | --- | --- | --- | --- |
|  |  | Variance random intercept | Variance random slope |  | Variance residual |
| **Outcomes** |  | σ^2^( a_i0_) | σ^2^( b_i_) |  | σ^2^( ε_ij_) |
| Hip extension strength (z-score) |  | 0.761 | 0.106 (b_i2_) |  | 0.832 |
| Hip flexion strength (z-score) |  | 1.101 |  |  | 0.570 |
| Hip abduction strength (z-score) |  | 0.991 |  |  | 0.721 |
| Knee extension strength (z-score) |  | 0.868 | 0.042 (b_i2_) |  | 0.584 |
| Knee flexion strength (z-score) |  | 3.843 | 0.032 (b_i_) |  | 0.277 |
| Plantar flexion strength (z-score) |  | 0.248 | 0.123 (b_i2_) |  | 0.901 |
| Dorsiflexion strength (z-score) |  | 7.747 | 0.158 (b_i1_) |  | 0.761 |
| Knee extension ROM (°) |  | 9.977 | 0.975 (b_i2_) |  | 2.476 |
| Knee extension ROM (z-score) |  | 0.406 | 0.039 (b_i2_) |  | 0.508 |
| Hamstrings ROM (°) |  | 114.260 |  |  | 72.385 |
| Hamstrings ROM (z-score) |  | 0.989 |  |  | 0.740 |
| Dorsiflexion ROM knee extended (°) |  | 35.935 |  |  | 17.838 |
| Dorsiflexion ROM knee extended (z-score) |  | 1.258 | 0.073 (b_i2_) |  | 0.735 |
| Dorsiflexion ROM knee flexed (°) |  | 37.930 |  |  | 18.381 |
| Dorsiflexion ROM knee flexed (z-score) |  | 0.845 |  |  | 0.448 |
| Rectus femoris CSA (z-score) |  | 1.242 |  |  | 0.471 |
| Medial gastrocnemius CSA (z-score) |  | 4.091 |  |  | 1.355 |
| Tibialis anterior CSA (z-score) |  | 1.589 |  |  | 0.317 |

The following symbols represent: σ^2^ =variance; a_i0_ = random intercept; b_i_ = random slope; b_i1_ = random slope for regression slope before breakpoint; b_i2_ = random slope for regression slope after breakpoint; ε_ij_ = measurement error.

CSA, cross-sectional area; DMD, Duchenne muscular dystrophy; ROM, range of motion;
