# Supplementary material for: Longitudinal trajectories of muscle impairments in growing boys with Duchenne muscular dystrophy
Source: PLoS One. 2025 Mar 18;20(3):e0307007. doi: 10.1371/journal.pone.0307007 (PMC11918350; doi:10.1371/journal.pone.0307007)
Supplement: S6 Table — The following symbols represent: σ2 = variance; ai0 = random intercept; bi1 = random slope for regression slope of age; εij = measurement error. CSA, cross-sectional area; DMD, Duchenne muscular dystrophy; ROM, range of motion. (DOCX) [file pone.0307007.s009.docx]

**S6 Table. Estimates of random-effect and residual covariance structure of linear mixed-effect models for the longitudinal trajectories of the muscle impairments with age for boys with DMD**

|  |  | **Random effects** | |  | **Residual** |
| --- | --- | --- | --- | --- | --- |
|  |  | Variance random intercept | Variance random slope |  | Variance residual |
| **Outcomes** |  | σ^2^( a_i0_) | σ^2^( b_i1_) |  | σ^2^( ε_ij_) |
| Hip extension strength (z-score) |  | 5.925 | 0.101 |  | 0.727 |
| Hip flexion strength (z-score) |  | 1.069 |  |  | 0.594 |
| Hip abduction strength (z-score) |  | 0.990 |  |  | 0.719 |
| Knee extension strength (z-score) |  | 2.246 | 0.026 |  | 0.352 |
| Knee flexion strength (z-score) |  | 3.843 | 0.032 |  | 0.277 |
| Plantar flexion strength (z-score) |  | 7.076 | 0.101 |  | 0.773 |
| Dorsiflexion strength (z-score) |  | 2.010 | 0.028 |  | 0.601 |
| Knee extension ROM (°) |  | 47.735 | 0.813 |  | 6.292 |
| Knee extension ROM (z-score) |  | 2.242 | 0.035 |  | 0.265 |
| Hamstrings ROM (°) |  | 114.260 |  |  | 72.385 |
| Hamstrings ROM (z-score) |  | 0.989 |  |  | 0.740 |
| Dorsiflexion ROM knee extended (°) |  | 34.937 |  |  | 18.328 |
| Dorsiflexion ROM knee extended (z-score) |  | 1.162 |  |  | 0.662 |
| Dorsiflexion ROM knee flexed (°) |  | 37.140 |  |  | 19.862 |
| Dorsiflexion ROM knee flexed (z-score) |  | 0.828 |  |  | 0.471 |
| Rectus femoris CSA (z-score) |  | 1.363 |  |  | 0.429 |
| Medial gastrocnemius CSA (z-score) |  | 3.847 |  |  | 1.397 |
| Tibialis anterior CSA (z-score) |  | 9.683 | 0.111 |  | 0.150 |

The following symbols represent: σ^2^ =variance; a_i0_ = random intercept; b_i1_ = random slope for regression slope of age; ε_ij_ = measurement error.

CSA, cross-sectional area; DMD, Duchenne muscular dystrophy; ROM, range of motion;
