# Supplementary figures and images for: Longitudinal trajectories of muscle impairments in growing boys with Duchenne muscular dystrophy
Source: PLoS One. 2025 Mar 18;20(3):e0307007. doi: 10.1371/journal.pone.0307007 (PMC11918350; doi:10.1371/journal.pone.0307007)

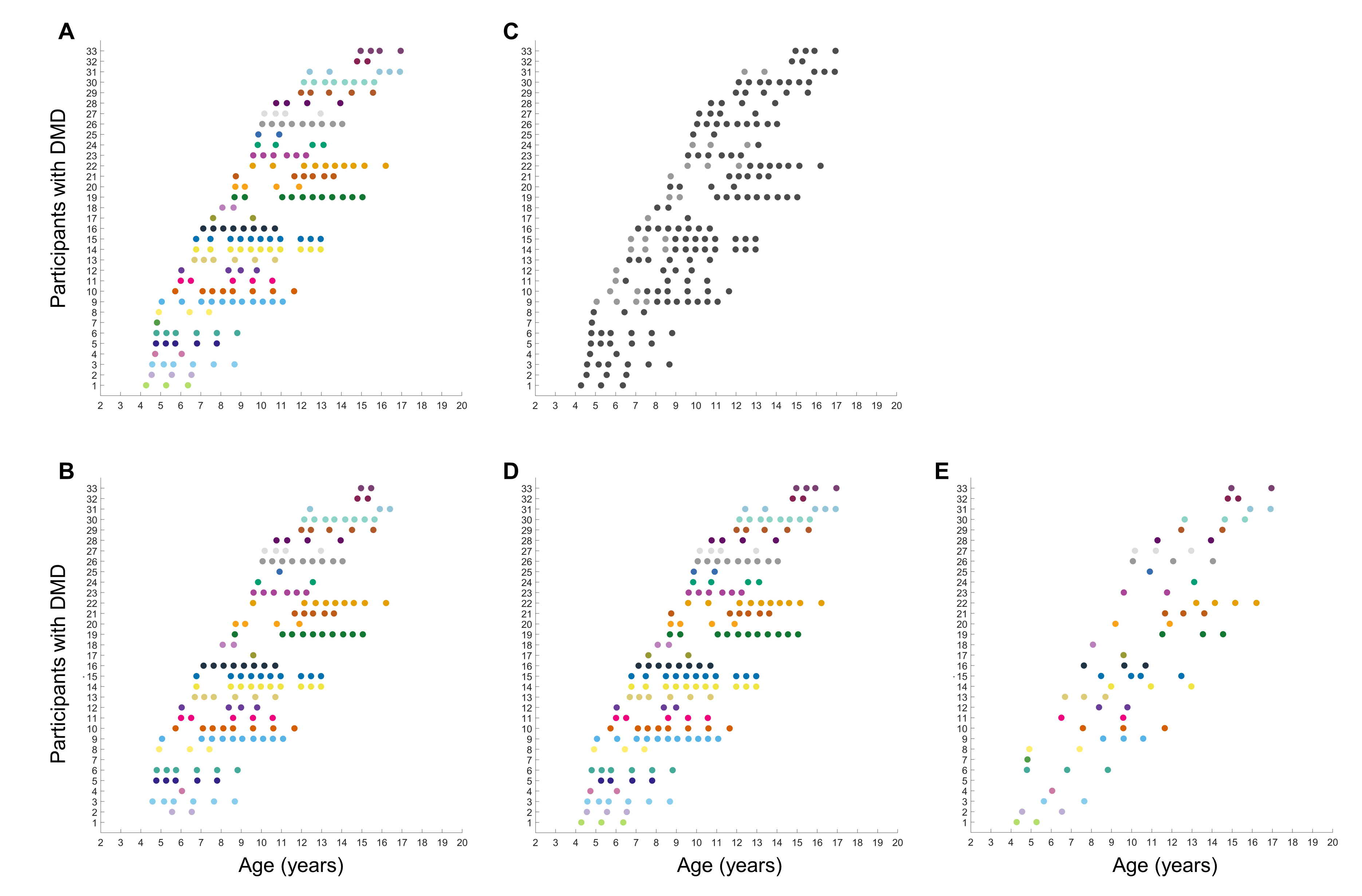

Supplement: S1 Fig — Every patient is represented in a different color. (A) Total database. (B) Strength dataset. (C) Total database, with measurements collected retrospectively shown in light gray and prospectively in dark gray. (D) ROM dataset. (E) Ultrasound dataset. DMD, Duchenne muscular dystrophy; ROM, range of motion. (TIF) [file pone.0307007.s010.tif]

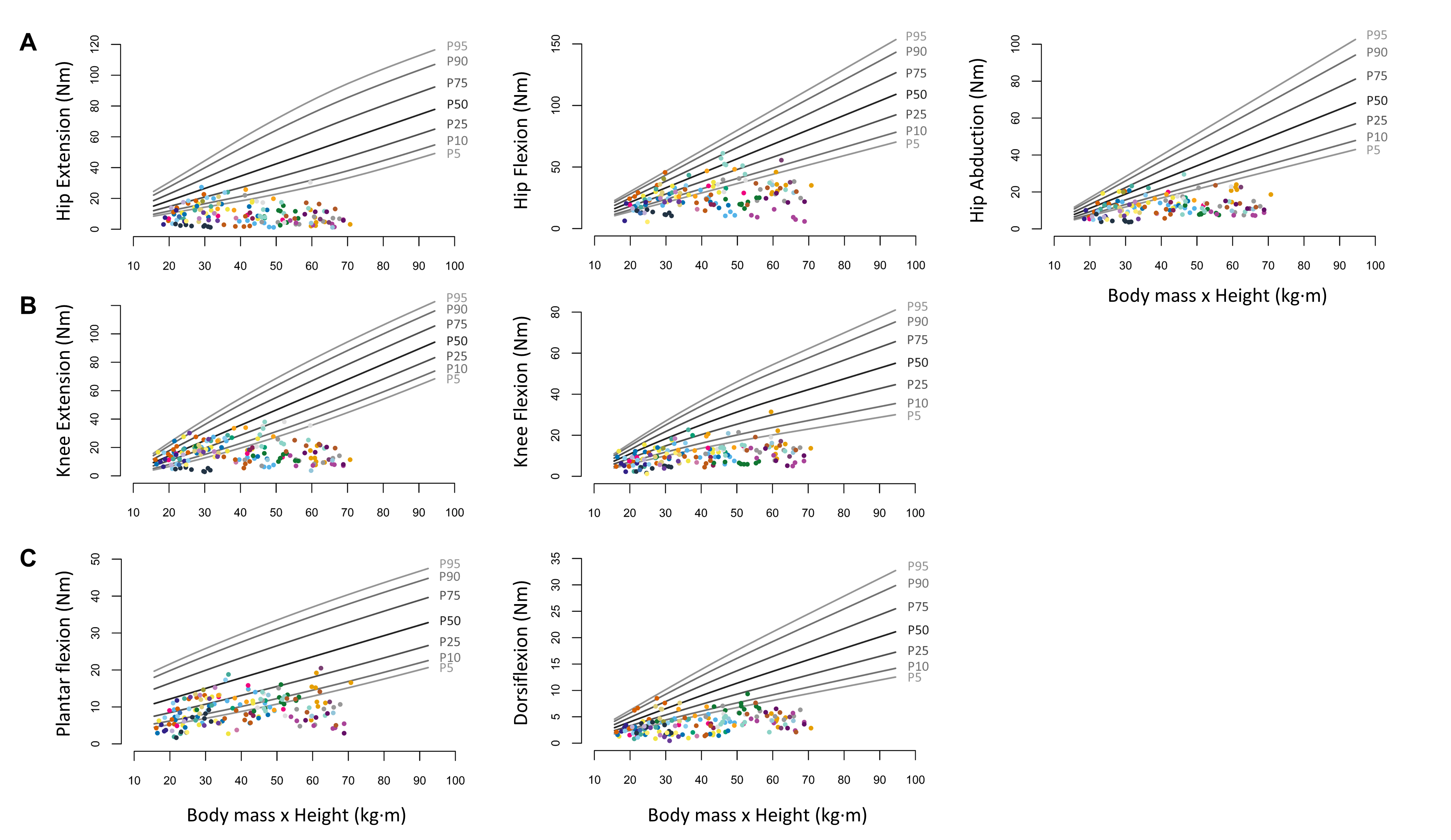

Supplement: S2 Fig — Estimated percentiles 5% (P5), 10% (P10), 25% (P25), 50% (P50), 75% (P75), 90% (P90), 95% (P95) of the change in absolute joint moments with increasing anthropometric values (i.e., body mass x height) of TD children are plotted. The colored dots indicate the absolute joint moments of the participants with DMD, with each participant represented in a different color. These curves were used to convert the absolute joint moments of the participants with DMD into unit-less z-scores, where P5 corresponds to a z-score of −1.645, P10 to −1.282, P25 to −0.675, P50 to 0, P75 to 0.675, P90 to 1.282, and P95 to 1.645. A z-score of 0 means that the absolute joint moment equals the median absolute joint moment of TD children. A z-score of +/−1 indicates that the absolute joint moment deviates by one standard deviation above or below the median of TD children, respectively. DMD, Duchenne muscular dystrophy; TD, typically developing. (TIF) [file pone.0307007.s011.TIF]

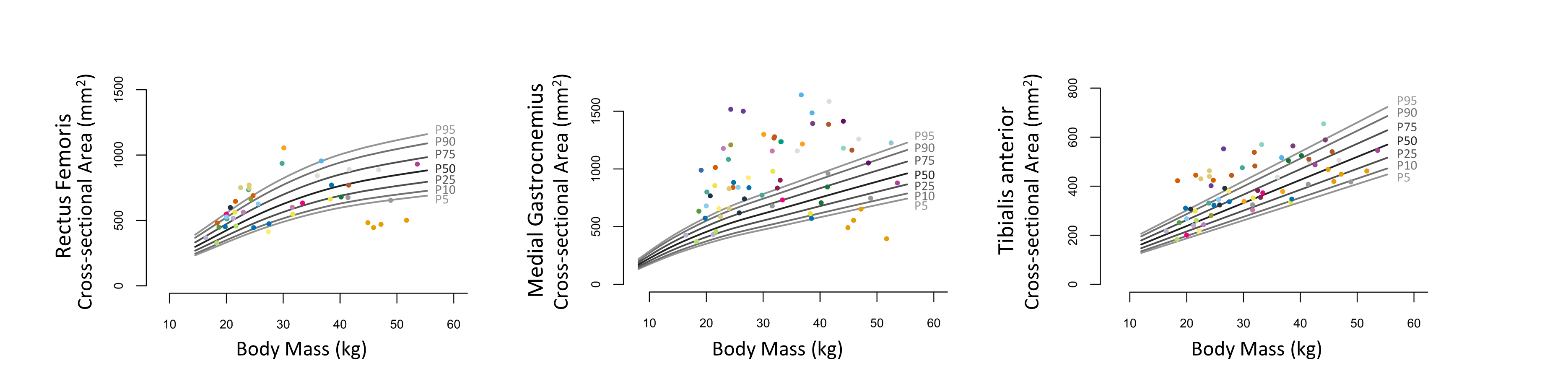

Supplement: S3 Fig — Estimated percentiles 5% (P5), 10% (P10), 25% (P25), 50% (P50), 75% (P75), 90% (P90), 95% (P95) of the change in absolute cross-sectional area with increasing body mass of TD children are plotted. The colored dots indicate the absolute cross-sectional areas of the participants with DMD, with each participant represented in a different color. These curves were used to convert the absolute cross-sectional areas of the participants with DMD into unit-less z-scores, where P5 corresponds to a z-score of -1.645, P10 to -1.282, P25 to -0.675, P50 to 0, P75 to 0.675, P90 to 1.282, and P95 to 1.645. A z-score of 0 means that the absolute cross-sectional area equals the median absolute cross-sectional area of TD children. A z-score of +/−1 indicates that the absolute cross-sectional area deviates by one standard deviation above or below the median of TD children, respectively. DMD, Duchenne muscular dystrophy; TD, typically developing. (TIF) [file pone.0307007.s012.tif]

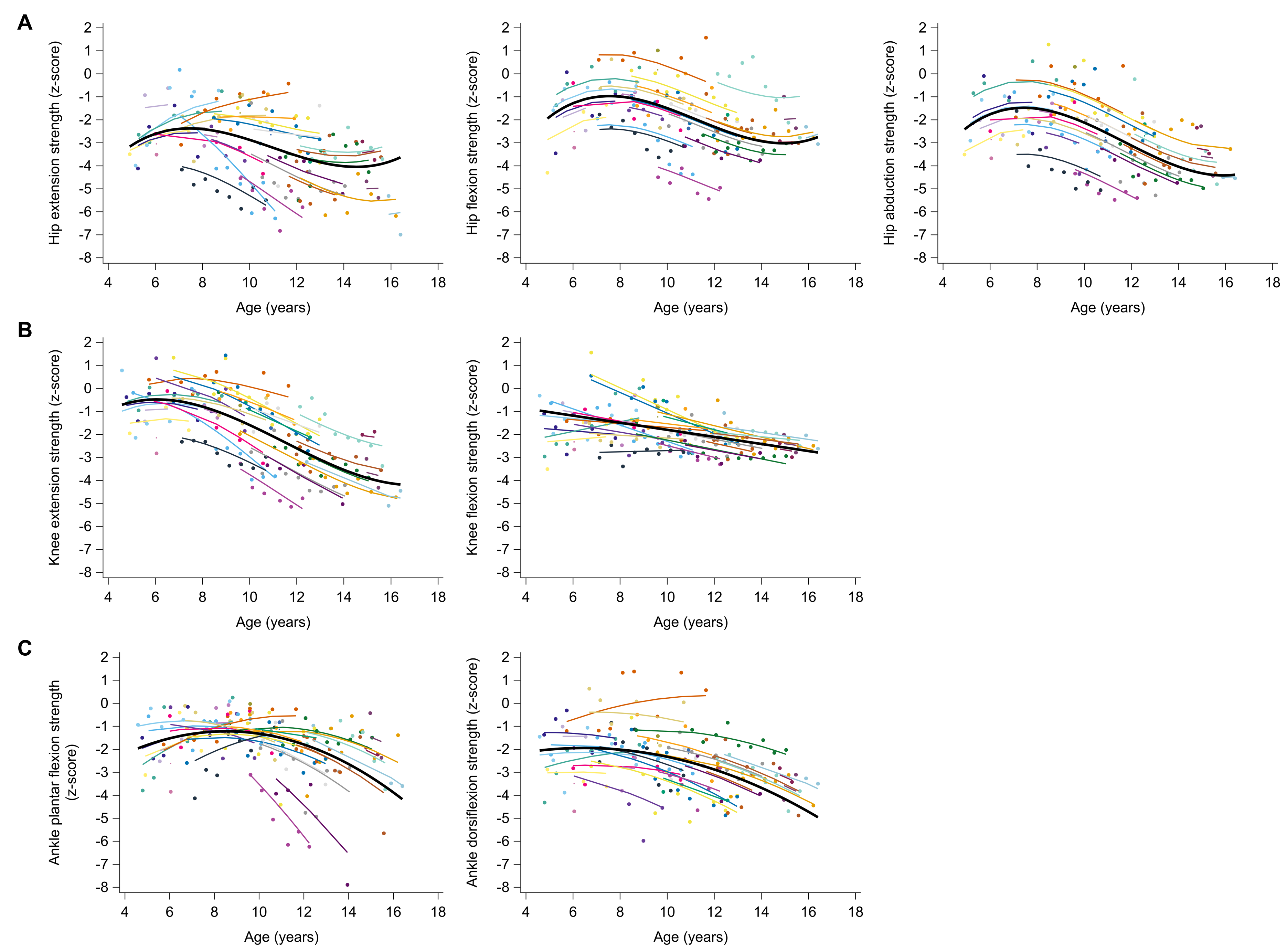

Supplement: S4 Fig — The average predicted trajectory (thick black line), the individual predicted profiles (thinner colored lines), and the actual observed outcomes (colored dots) are displayed. Each color represents one patient with DMD. The estimates for the fixed effects are given in S1 Table. DMD, Duchenne muscular dystrophy. (TIF) [file pone.0307007.s013.TIF]

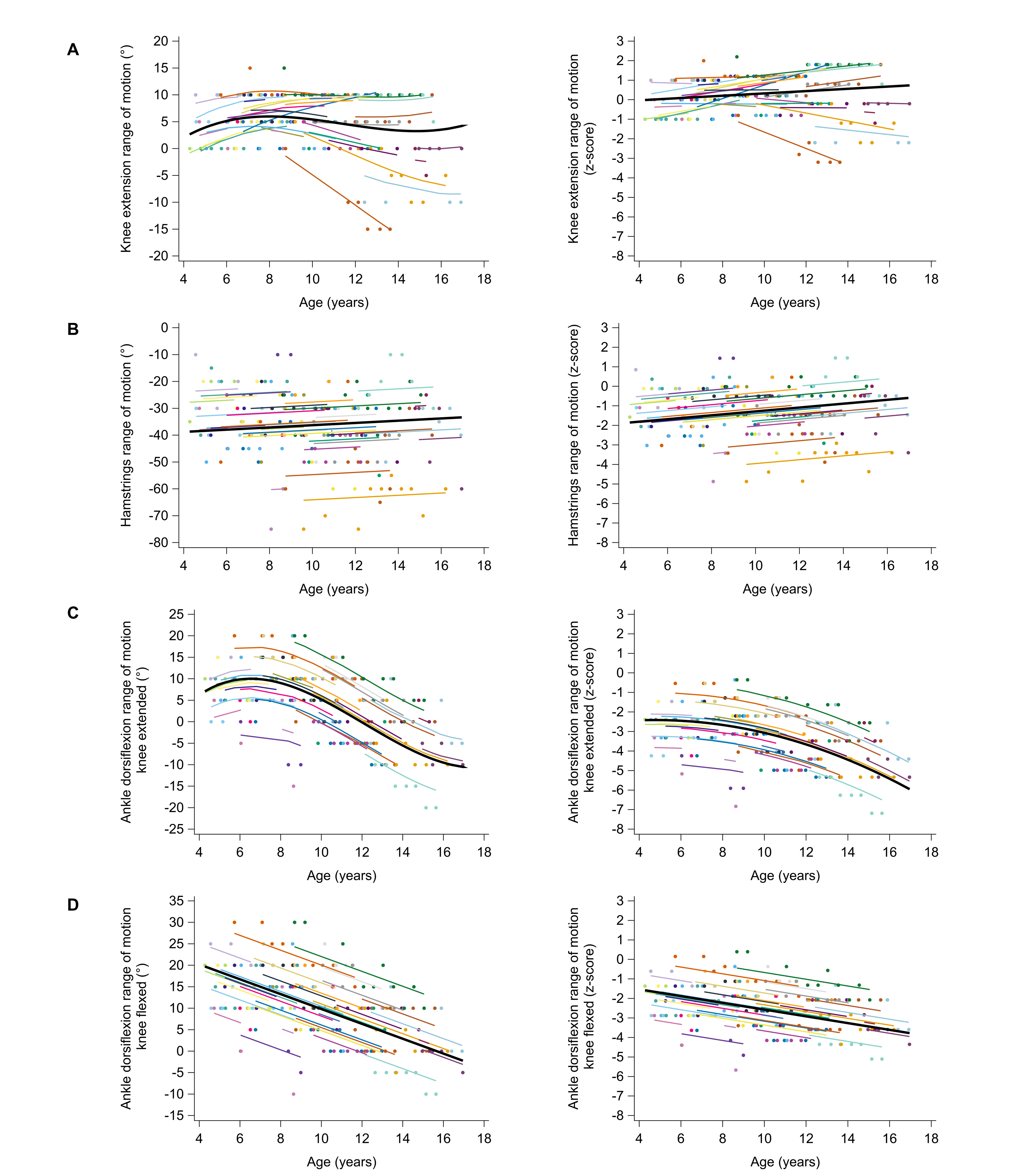

Supplement: S5 Fig — The average predicted trajectory (thick black line), the individual predicted profiles (thinner colored lines), and the actual observed outcomes (colored dots) are displayed. Each color represents one patient with DMD. The estimates for the fixed effects are given in S2 Table. DMD, Duchenne muscular dystrophy; ROM, range of motion. (TIF) [file pone.0307007.s014.TIF]

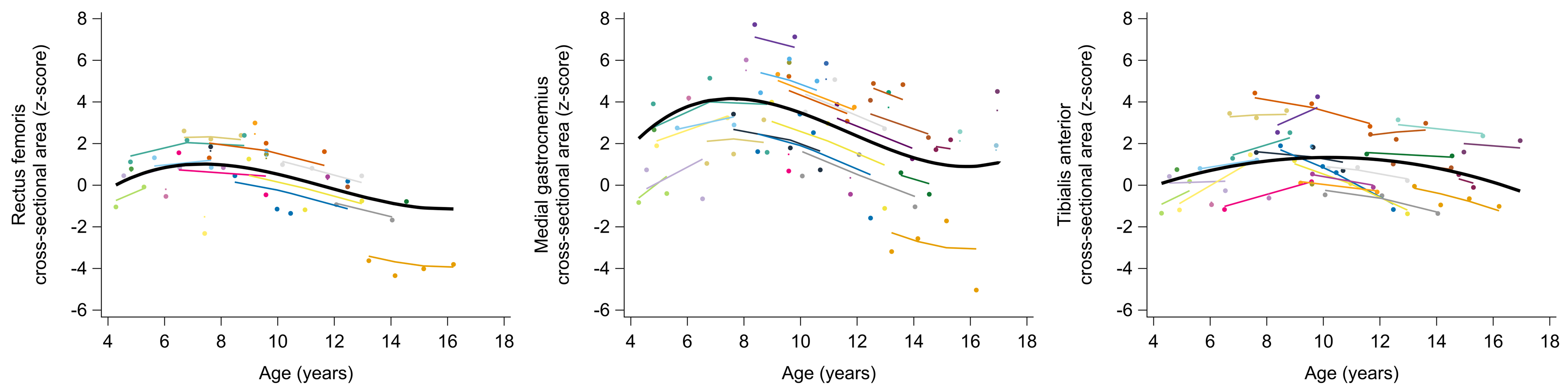

Supplement: S6 Fig — The average predicted trajectory (thick black line), the individual predicted profiles (thinner colored lines), and the actual observed outcomes (colored dots) are displayed. Each color represents one patient with DMD. The estimates for the fixed effects are given in S3 Table. DMD, Duchenne muscular dystrophy. (TIF) [file pone.0307007.s015.TIF]

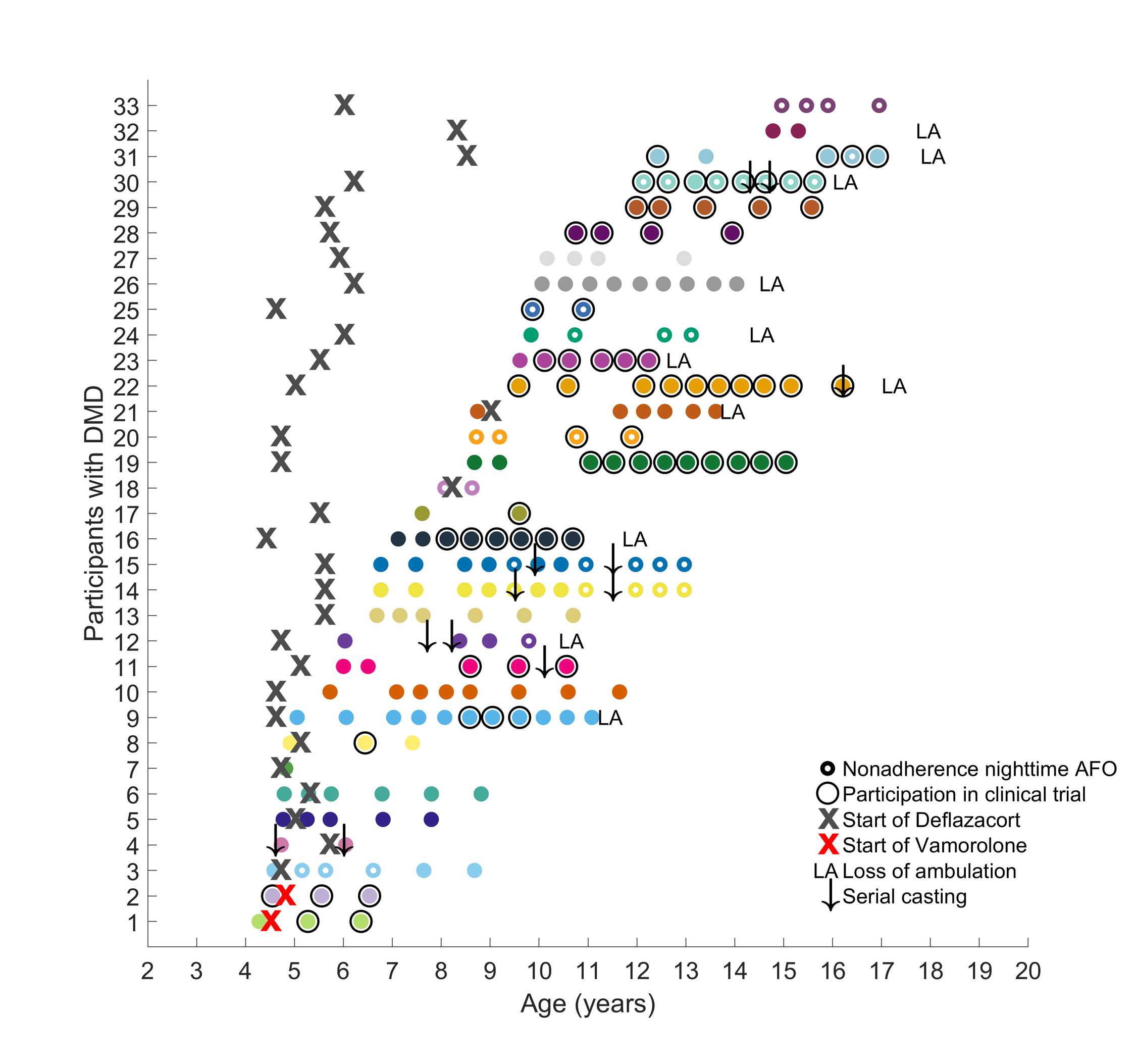

Supplement: S7 Fig — Each color represents one patient with DMD. AFO, ankle foot orthosis; DMD, Duchenne muscular dystrophy; LA, loss of ambulation. (TIF) [file pone.0307007.s016.tif]

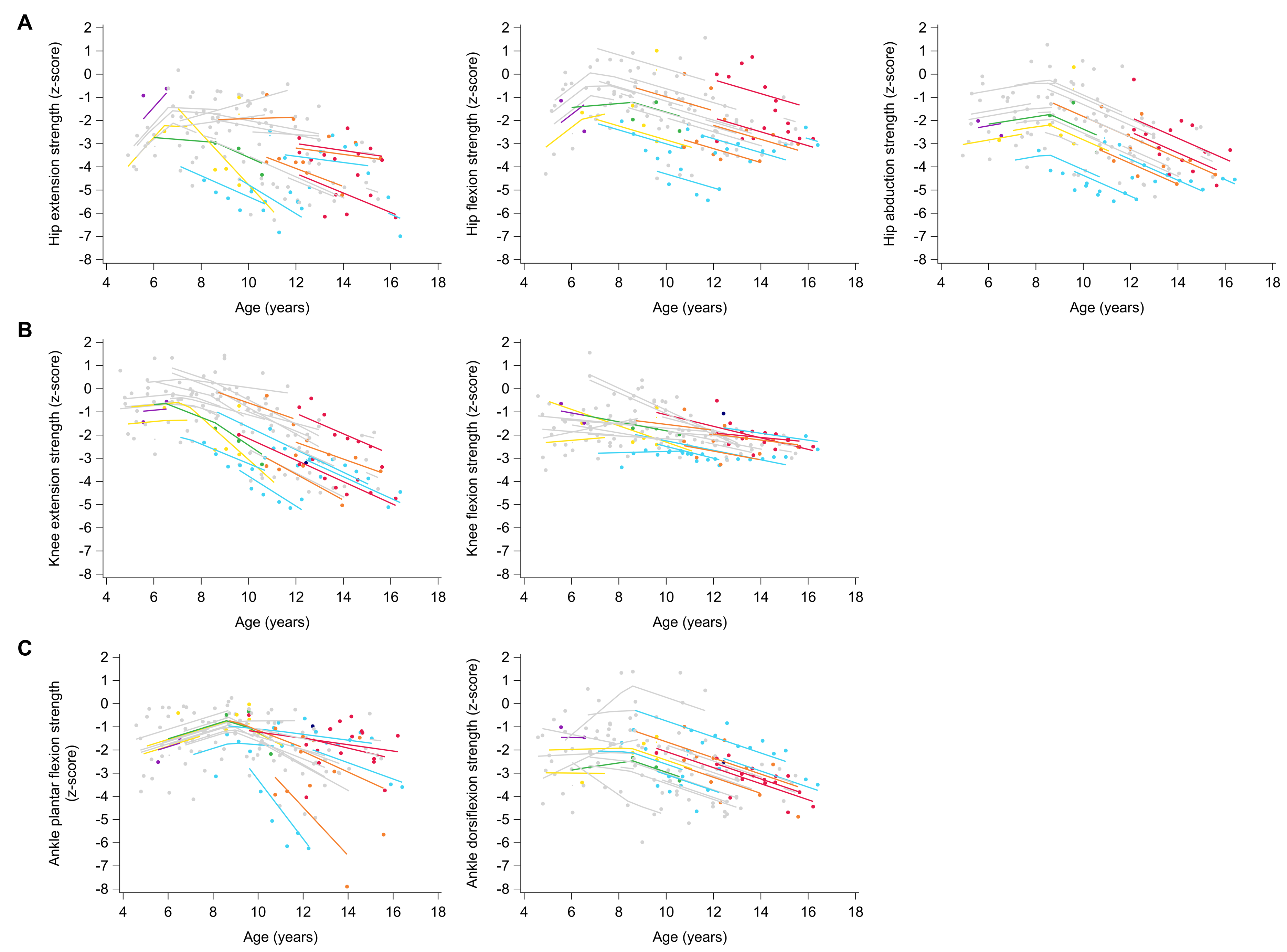

Supplement: S8 Fig — The observed actual values during clinical trial participation are color-coded: red for Ataluren, orange for Exon-skipping 45, yellow for Exon-skipping 51, green for Exon-skipping 53, blue for Givinostat, navy blue for Tadalafil, and purple for Vamorolone. If a boy participated in a clinical trial at any point during follow-up, his entire predicted profile is displayed in the corresponding color. For boys who did not participate in any clinical trials, both the observed values and predicted profiles are shown in gray. No major conclusions can be drawn from this data exploration due to significant variability among children within the same trials. (TIF) [file pone.0307007.s017.TIF]

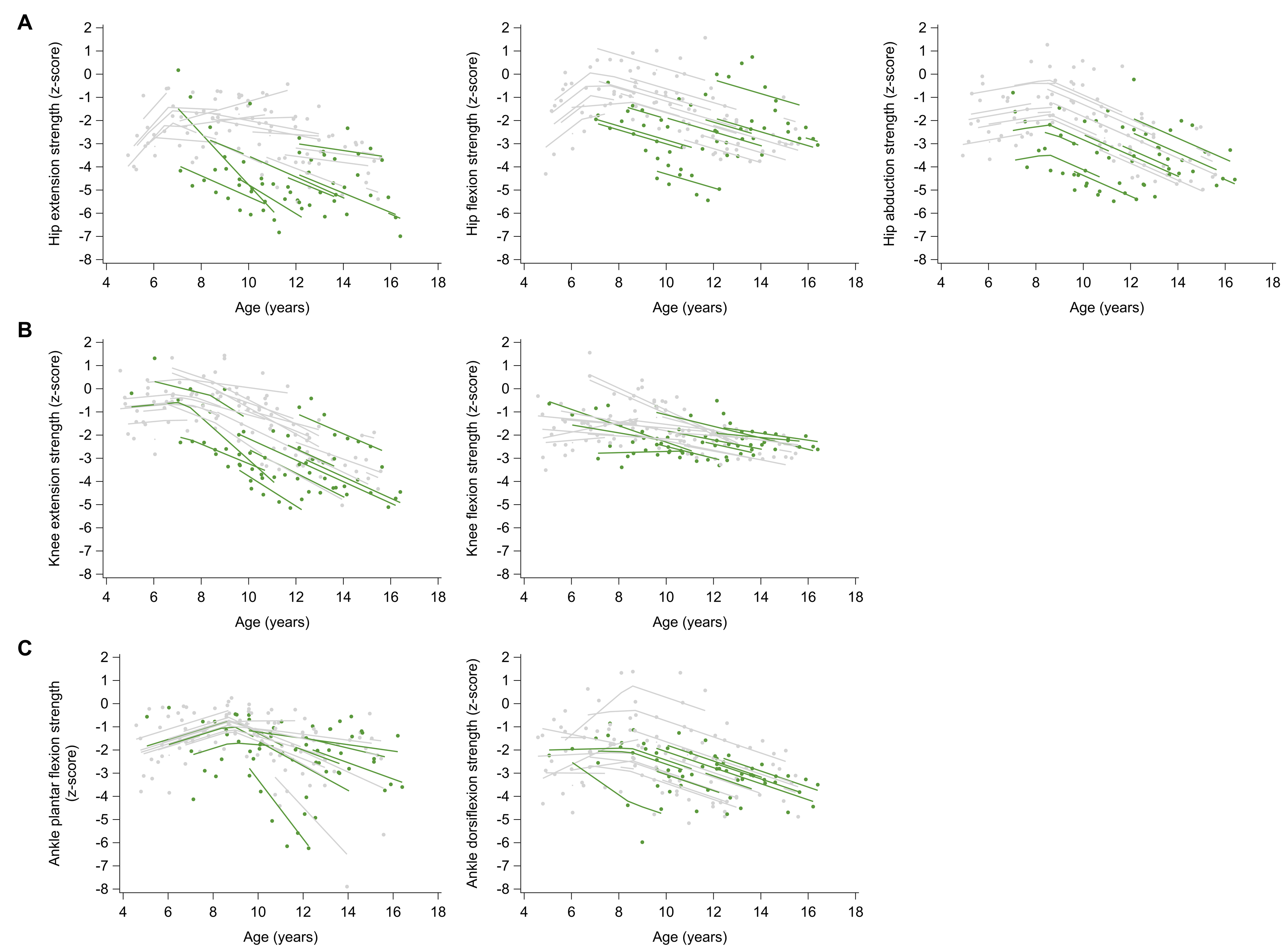

Supplement: S9 Fig — Boys who lost ambulation overall showed lower z-scores, especially for hip and knee extension strength. (TIF) [file pone.0307007.s018.TIF]

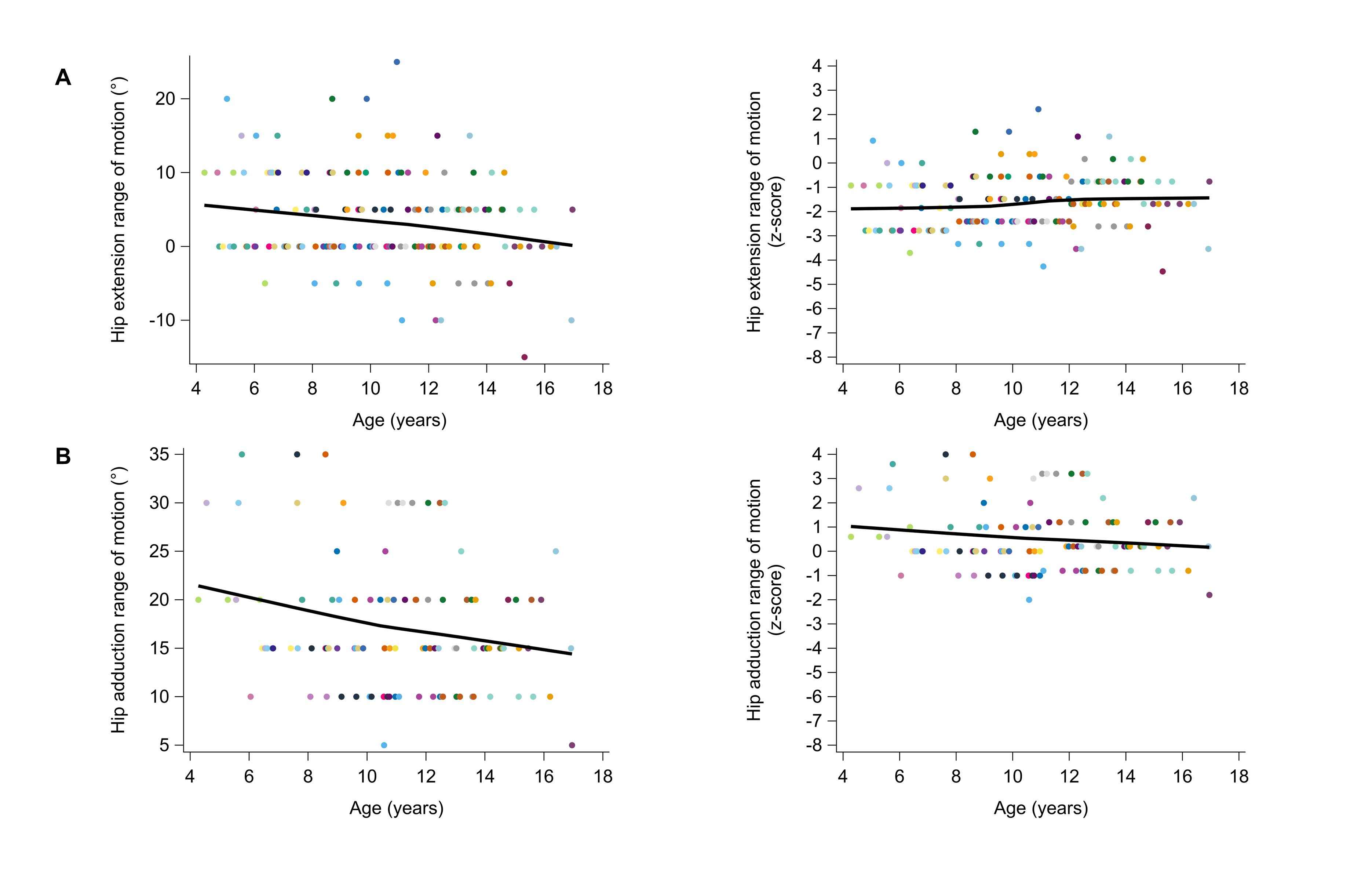

Supplement: S10 Fig — The Loess regression (thick black line) and the actual observed outcomes (colored symbols) are displayed. Each color represents one patient with DMD. DMD, Duchenne muscular dystrophy; ROM, range of motion. (TIF) [file pone.0307007.s019.TIF]

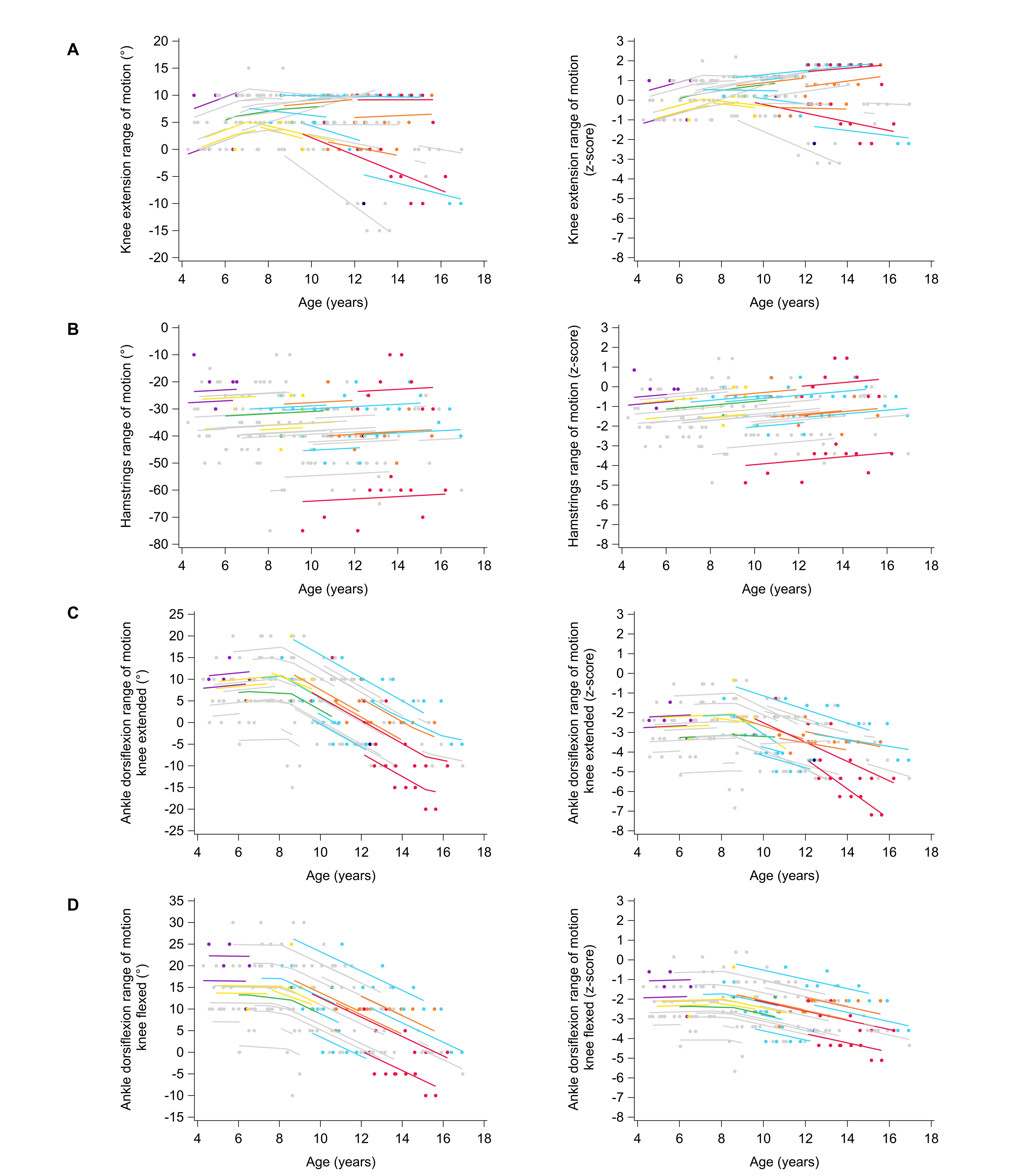

Supplement: S11 Fig — The observed actual values during clinical trial participation are color-coded: red for Ataluren, orange for Exon-skipping 45, yellow for Exon-skipping 51, green for Exon-skipping 53, blue for Givinostat, navy blue for Tadalafil, and purple for Vamorolone. If a boy participated in a clinical trial at any point during follow-up, his entire predicted profile is displayed in the corresponding color. For boys who did not participate in any clinical trials, both the observed values and predicted profiles are shown in gray. No major conclusions can be drawn from this data exploration due to significant variability among children within the same trials. ROM, range of motion. (TIF) [file pone.0307007.s020.TIF]

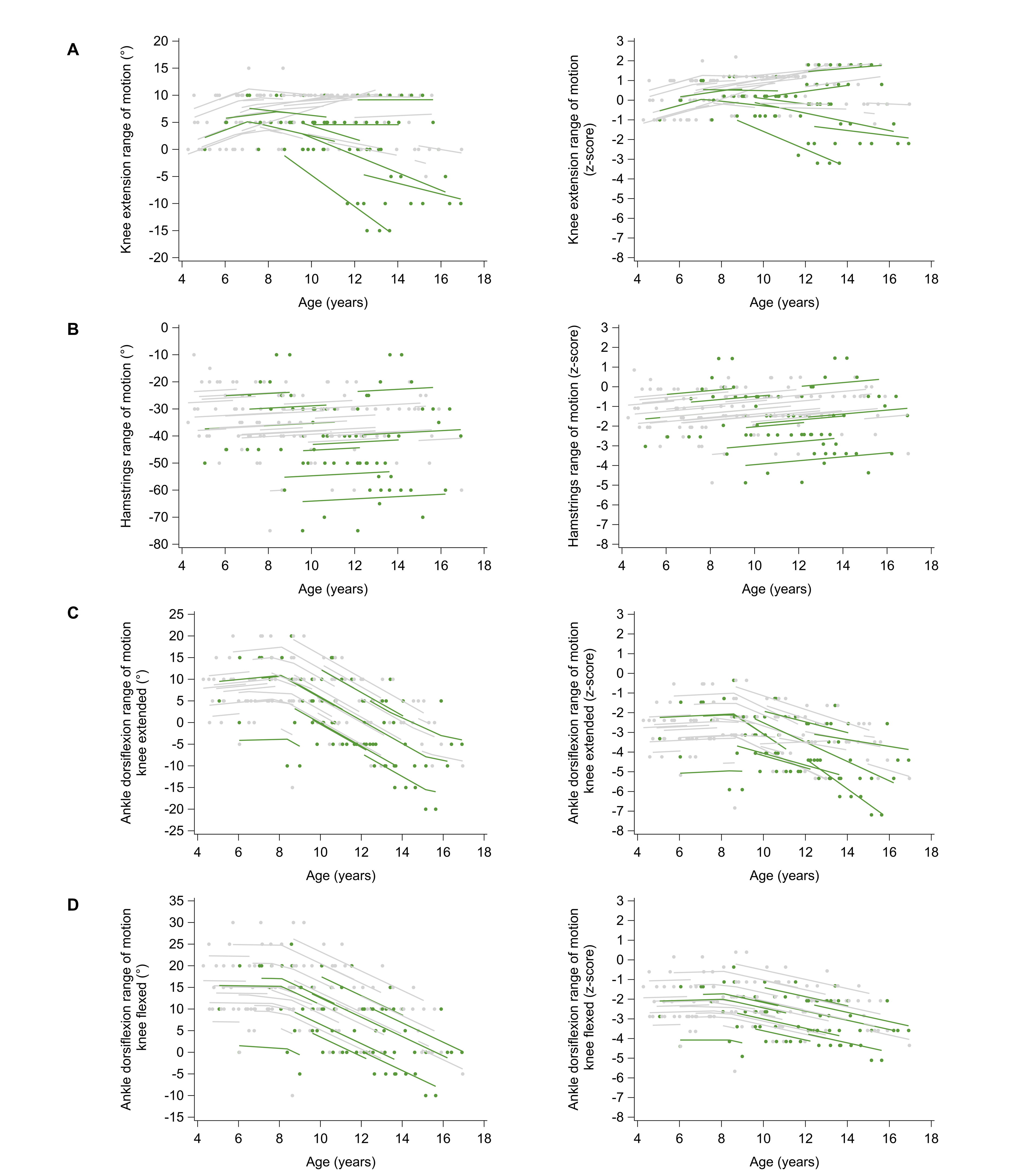

Supplement: S12 Fig — Boys who lost ambulation overall showed lower z-scores, especially for knee extension ROM. ROM, range of motion. (TIF) [file pone.0307007.s021.TIF]

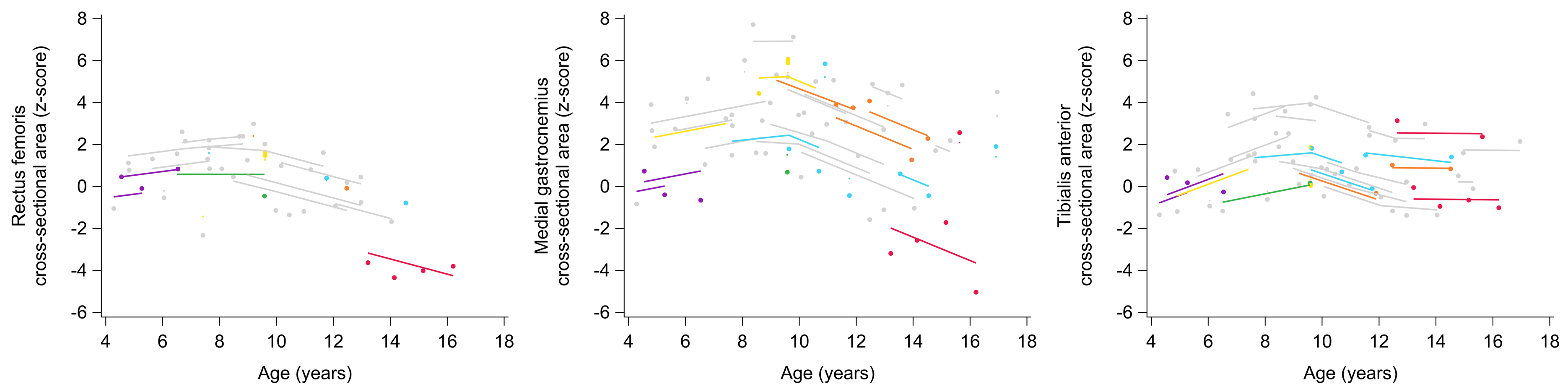

Supplement: S13 Fig — The observed actual values during clinical trial participation are color-coded: red for Ataluren, orange for Exon-skipping 45, yellow for Exon-skipping 51, green for Exon-skipping 53, blue for Givinostat, navy blue for Tadalafil, and purple for Vamorolone. If a boy participated in a clinical trial at any point during follow-up, his entire predicted profile is displayed in the corresponding color. For boys who did not participate in any clinical trials, both the observed values and predicted profiles are shown in gray. No major conclusions can be drawn from this data exploration due to significant variability among children within the same trials. (TIF) [file pone.0307007.s022.TIF]

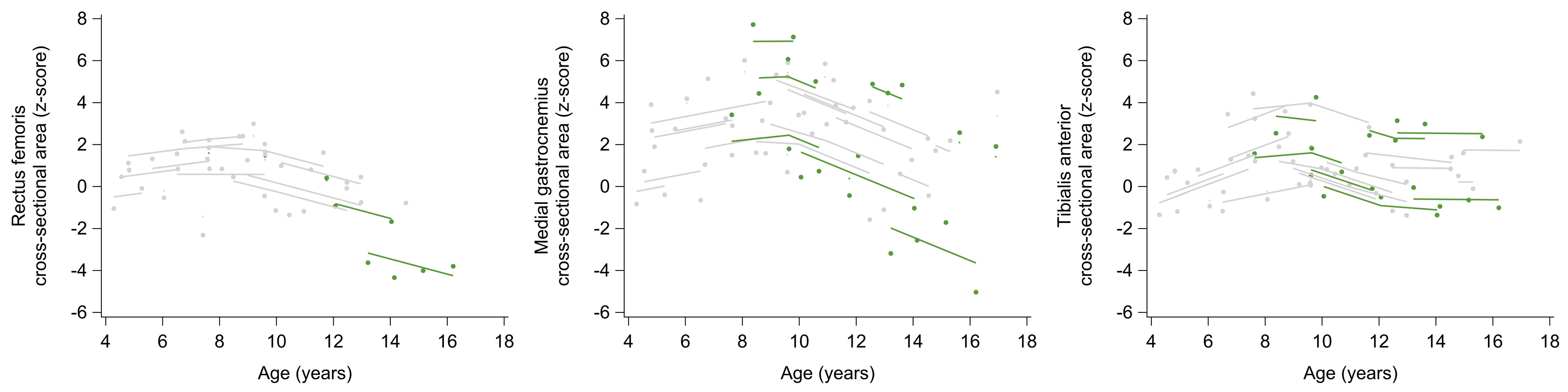

Supplement: S14 Fig — No major conclusion can be drawn. It was often not feasible to process the data for the m. rectus femoris in more severely affected children, resulting in data exclusion and making the dataset not fully generalizable. (TIF) [file pone.0307007.s023.TIF]
